# Linking Stoichiometry, Chemical Pressure, and Domain Structure in the van der Waals Ferrielectric CuInP_2_S_6_

**DOI:** 10.3390/nano16211336

**Published:** 2026-10-22

**Authors:** Dávid Gál, Sándor Lenk, István Rigó, Miklós Veres, László Péter, Anton Kohutych, Konstantin Glukhov, Nyomó Dominika, Alexander Molnar

**Affiliations:** 1 HUN-REN Wigner Research Centre for Physics, Konkoly-Thege Miklós út 29-33, 1121 Budapest, Hungary; gal.david@wigner.hun-ren.hu (D.G.); rigo.istvan@wigner.hu (I.R.); veres.miklos@wigner.hun-ren.hu (M.V.); peter.laszlo@wigner.hun-ren.hu (L.P.); 2 Institute of Physics, University of Pécs, Ifjúság útja. 6, 7624 Pécs, Hungary; 3 Department of Atomic Physics, Institute of Physics, Budapest University of Technology and Economics, Műegyetem rkp. 3, 1111 Budapest, Hungary; lenk.sandor@ttk.bme.hu; 4 Department of the Physics of Semiconductors, Uzhhorod National University, 54 Voloshina Str., 88000 Uzhhorod, Ukraine; anton.kohutych@uzhnu.edu.ua (A.K.); konstantin.glukhov@uzhnu.edu.ua (K.G.);

**Keywords:** CuInP_2_S_6_, van der Waals ferrielectrics, stoichiometry, phase transition, dielectric properties, domain structure

## Abstract

The ferrielectric properties of layered CuInP_2_S_6_ (CIPS) are highly sensitive to stoichiometry and growth-induced defects. Here we systematically compare crystals grown by chemical vapor transport and the Bridgman–Stockbarger method with Cu- and In-enriched samples, combining compositional analysis, dielectric spectroscopy, uniaxial-pressure measurements, Raman spectroscopy, piezoresponse force microscopy and first-principles calculations. The transition temperature increases systematically from 307 K (Bridgman) to 312 K (Cu-enriched), 317 K (gas transport) and 322 K (In-enriched). In enrichment is consistent with spontaneous CIPS/In_4_/_3_P_2_S_6_ phase segregation that generates effective positive chemical pressure, stabilizing the ferrielectric state and producing a strongly biased, pinned domain configuration. Cu enrichment produces the opposite response—lower TC, enhanced dielectric loss and a fragmented domain morphology—characteristic of a negative-pressure-like perturbation of the Cu-ion energy landscape. Landau–Ginzburg–Devonshire analysis and density functional calculations provide a unified microscopic picture based on Cu-ion configurational freedom and interlayer coupling. Growth defects act as an independent source of domain-wall pinning. Together, these results establish stoichiometry and synthesis conditions as practical tools for engineering ferrielectric order in van der Waals CIPS.

## 1. Introduction

Layered metal thio- and selenophosphates have attracted considerable attention because their low-dimensional crystal structures combine van der Waals bonding with a broad range of functional electronic and ferroic properties [1,2]. Among these materials, CuInP_2_S_6_ (CIPS) is particularly interesting as a layered ferrielectric that retains spontaneous polarization down to the nanoscale [3,4]. This combination of reduced dimensionality and switchable polarization has motivated extensive research toward CIPS-based ferroelectric field-effect transistors [5], negative-capacitance devices [6], memristors [7], and neuromorphic and optoelectronic synaptic devices [8,9]. However, the relatively low ferrielectric transition temperature, *T_C_* ≈ 315 K, limits the temperature range of the polar state, while the pronounced sensitivity of CIPS properties to growth conditions and stoichiometry presents both a reproducibility challenge and an opportunity for controlled materials engineering.

**Figure 1:**
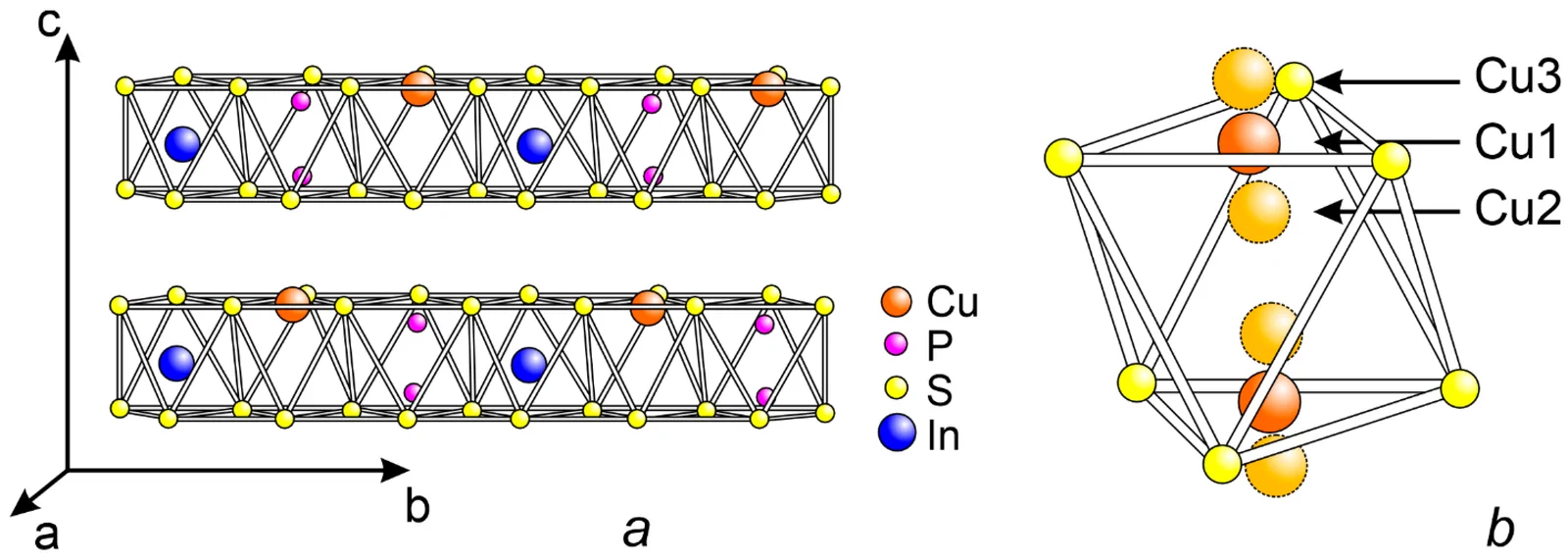
(**a**) Layered crystal structure of CuInP_2_S_6_ viewed along the a axis. (**b**) Local sulfur coordination showing the Cu1, Cu2, and Cu3 positions [11].

CuInP_2_S_6_ has a layered structure in which Cu, In, and P–P pairs occupy octahedral sites within a sulfur framework [10]. Individual layers are weakly coupled by van der Waals interactions, making the structure particularly sensitive to changes in interlayer spacing and cation distribution. The ferrielectric ordering is closely associated with the displacement and redistribution of Cu^+^ ions among several energetically nonequivalent positions (Cu1, Cu2, and Cu3; Figure 1) [11]. Above *T_C_*, thermally activated Cu-ion hopping between available sites contributes to dynamic disorder, whereas cooling through the transition preferentially stabilizes off-center Cu positions together with oppositely directed displacements of the In^3+^ sublattice. The resulting partially compensating polar sublattices produce the ferrielectric state. Consequently, modifications of the Cu-ion potential landscape, site occupancy, or interlayer coupling can strongly affect the transition temperature and polarization behavior of CIPS.

An important route for modifying the properties of CIPS is deviation from stoichiometry. Dziaugys et al. [12] demonstrated that In enrichment increases the phase-transition temperature, with *T_C_* reaching approximately 330 K in crystals grown with excess In_2_S_3_. Subsequent studies showed that In-rich, Cu-deficient CIPS undergoes spontaneous chemical phase separation rather than forming a homogeneous solid solution [13,14]. The resulting structure consists of coexisting ferrielectric CuInP_2_S_6_ and centrosymmetric In_4_/_3_P_2_S_6_ (IPS) chemical domains within individual layers. Importantly, the lateral dimensions and spatial organization of these domains depend strongly on the thermal history, providing a means of controlling the mesoscale chemical architecture of CIPS/IPS heterostructures [13,14].

The formation of CIPS/IPS chemical domains has important structural and functional consequences. With decreasing Cu content, the transition temperature of the CIPS component increases despite the reduced fraction of the ferrielectric phase [14]. X-ray diffraction reveals a systematic composition dependence of the interlayer spacing, which increases from approximately 6.5054 Å in In_4_/_3_P_2_S_6_ to 6.5146 Å in CuInP_2_S_6_ [14]. More importantly, the coexistence of the two phases produces substantial and opposite strains in the CIPS and IPS regions [14,15]. This mutual strain provides a physical basis for interpreting the effect of In enrichment on the CIPS phase as an effective positive chemical pressure. The interfaces between the chemical phases also modify Cu-ion transport and conductivity [16], while the observed coupling between CIPS/IPS chemical domains and ferrielectric domains demonstrates that compositional heterogeneity, strain, and polarization organization are intrinsically interconnected [15].

The strain effects associated with compositional heterogeneity suggest a close connection between stoichiometry and mechanical pressure. External pressure is known to strongly modify the ferrielectric transition in CIPS [17,18,19,20]. In the low-pressure regime most relevant to the present work, hydrostatic compression shifts *T_C_* toward higher temperatures, with a reported pressure coefficient *dT_C_*/*dp* ≈ 210 K GPa^−1^ [18,19]. A similar trend is observed under uniaxial compression, where *T_C_* increases to approximately 324 K at 0.06 GPa [20]. Hydrostatic pressure also modifies the Cu-ion distribution and can substantially enhance the spontaneous polarization through the increased occupation of interlayer Cu sites [21]. These observations emphasize the strong coupling between mechanical compression, Cu-site occupancy, and ferrielectric ordering, and are consistent with the order–disorder character of the phase transition in CIPS.

At higher pressures, the response of CIPS becomes substantially more complex, involving pronounced changes in Cu coordination, interlayer geometry, lattice dynamics, and eventually crystal symmetry [17,22]. Pressure-induced changes in Cu-related vibrational modes were observed by Raman spectroscopy [17], while the assignment of these modes to Cu-ion dynamics is consistent with earlier temperature-dependent Raman studies [23]. These transformations further emphasize the exceptional sensitivity of the CIPS lattice to the Cu-ion configuration; however, the high-pressure structural regimes lie outside the effective chemical-pressure analogy considered in the present work. Here, the relevant comparison is restricted primarily to the low-pressure regime, in which CIPS remains in the ferrielectric phase and compression modifies Cu-site occupancy and interlayer coupling without inducing the major structural transformations characteristic of higher pressures [24].

Taken together, these studies reveal a striking parallel between the compositional and mechanical control of ferrielectricity in CIPS. External compression increases *T_C_*, modifies Cu-site occupancy, and affects Cu-ion mobility [18,19,20,21,24], whereas In-rich, Cu-deficient CIPS is known to form CIPS/IPS chemical heterostructures in which lattice mismatch generates compressive strain in the CIPS regions and is likewise accompanied by an increase in *T_C_* [13,14,15]. Conversely, the systematic increase in interlayer spacing with increasing Cu content [14] suggests that Cu enrichment may drive the lattice in the opposite direction, producing a tensile or negative-pressure-like response. These observations raise the possibility that deviations from stoichiometry and externally applied pressure act on common microscopic degrees of freedom associated with Cu-ion configuration and interlayer coupling. However, a systematic experimental connection between stoichiometry, effective pressure, lattice dynamics, dielectric response, and ferrielectric domain organization in CuInP_2_S_6_ has not yet been established.

In this work, we systematically investigate how crystal growth and controlled deviations from stoichiometry modify the ferrielectric properties of CuInP_2_S_6_. Crystals grown by chemical vapor transport and the Bridgman–Stockbarger method are compared with Cu- and In-enriched samples using compositional analysis, dielectric spectroscopy, Raman spectroscopy, and piezoresponse force microscopy. We show that In enrichment produces a response characteristic of effective positive chemical pressure, whereas Cu enrichment drives the system toward a negative-pressure-like state. The resulting changes in *T_C_*, dielectric response, lattice dynamics, and domain morphology can be consistently understood in terms of modifications of the Cu-ion potential landscape, site occupancy, and interlayer coupling. This establishes a unified physical framework linking growth conditions and stoichiometry to chemical pressure and, ultimately, to the mesoscale ferrielectric domain structure of CuInP_2_S_6_.

## 2. Materials and Methods

### 2.1. Crystal Growth and Sample Preparation

CuInP_2_S_6_ crystals were first synthesized by Nitsche using chemical vapor transport reactions [25], and the subsequent development of melt-growth approaches, including the Bridgman–Stockbarger method [26], enabled the preparation of substantially larger bulk crystals. In the present work, single crystals were grown by both chemical vapor transport (CVT) and directional crystallization from the melt. In addition to nominally stoichiometric CuInP_2_S_6_, crystals with controlled deviations from stoichiometry were prepared from starting mixtures enriched with either Cu_2_S or In_2_S_3_.

For CVT growth, the starting materials were sealed in evacuated quartz ampoules approximately 15 cm in length, with CuI used as the transport agent. The temperature of the hot zone was maintained at 913–943 K and that of the cold zone at 903–913 K, corresponding to a temperature difference of 20–40 K. The resulting crystals formed thin platelets whose major faces were parallel to the structural layers; the layer normal was approximately along c*. In addition to nominally stoichiometric material, Cu- and In-enriched crystals were grown from starting mixtures containing 90 wt.% CuInP_2_S_6_ and 10 wt.% Cu_2_S, and 90 wt.% CuInP_2_S_6_ and 10 wt.% In_2_S_3_, respectively. Thus, the coefficients 0.9 and 0.1 used to denote these starting mixtures correspond to mass fractions rather than molar fractions.

**Figure 2:**
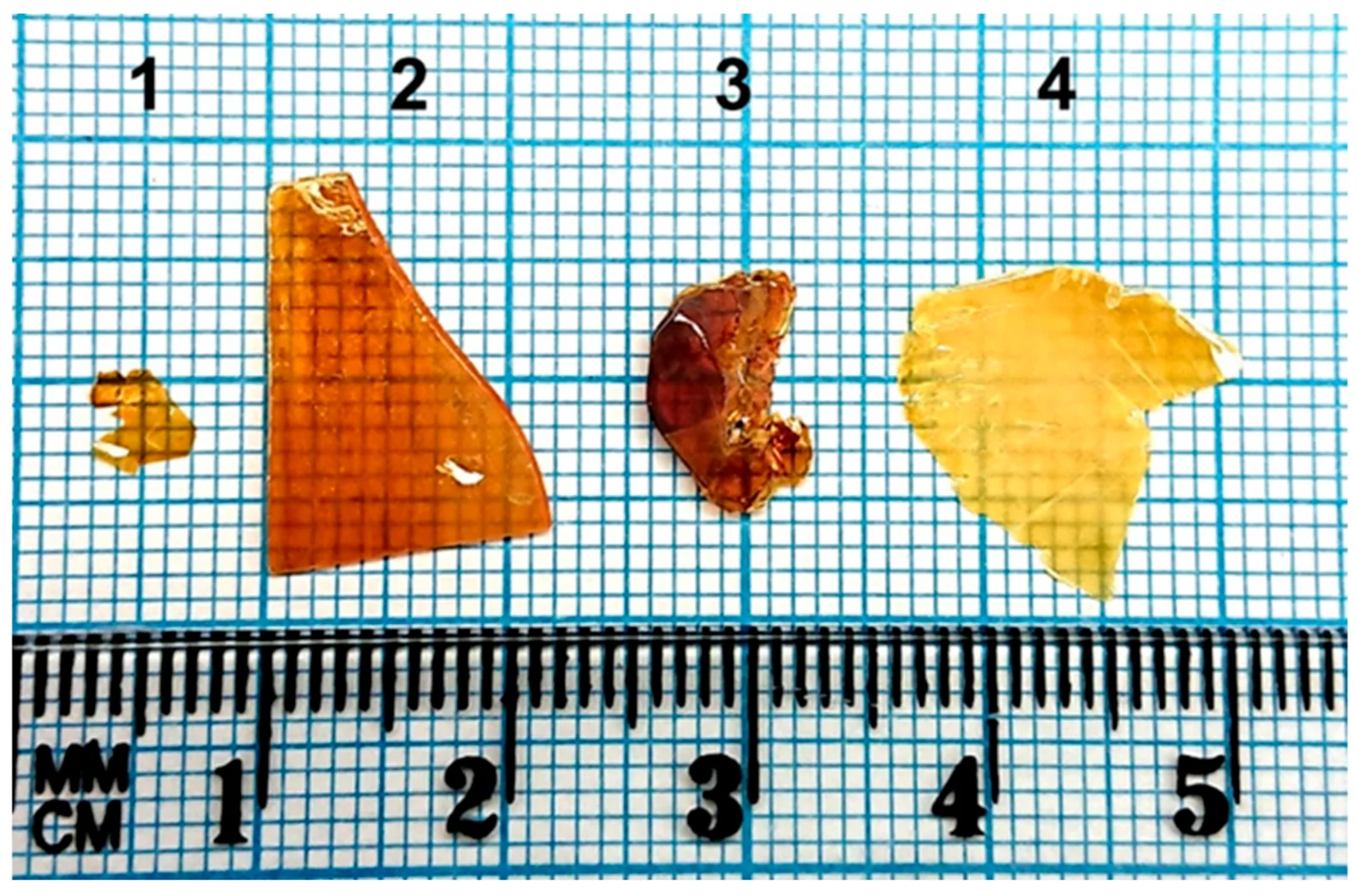
Photographs of CuInP_2_S_6_ samples obtained using different growth methods and starting compositions: 1—gas transport-grown CuInP_2_S_6_, 2—Bridgman–Stockbarger-grown CuInP_2_S_6_, 3—Cu-enriched CuInP_2_S_6_ + Cu_2_S, and 4—In-enriched CuInP_2_S_6_ + In_2_S_3_.

Bulk CuInP_2_S_6_ crystals were also prepared by directional crystallization from the melt using the Bridgman–Stockbarger method in a two-zone furnace [27]. The melt and annealing zones were maintained at 1150 and 870 K, respectively, with a temperature gradient of approximately 3 K mm^−1^ in the crystallization region. The crystallization front was advanced at 2.5 mm day^−1^, followed by cooling at approximately 200 K day^−1^. The starting material was synthesized from high-purity elemental components in stoichiometric proportions in quartz ampoules evacuated to approximately 0.013 Pa. The resulting ingots had a diameter of approximately 14 mm and a cylindrical length of 20–25 mm. Representative photographs of the four crystal types are shown in Figure 2.

### 2.2. Structural and Compositional Characterization

Surface morphology and chemical composition were examined using a TESCAN MIRA 3 (TESCAN, Brno, Czech Republic) scanning electron microscope equipped with an EDAX energy-dispersive X-ray spectroscopy (EDS) system (EDAX Inc., Mahwah, NJ, USA). Spectra were acquired and processed using EDAX APEX software (version 3.1). The samples were mounted on double-sided conductive carbon tape (Ted Pella, Redding, CA, USA). The compositions reported in Table 1 were obtained by averaging 5–7 local measurements acquired at different positions. Representative SEM images, quantitative results, and EDS spectra are provided in Figures S1–S4 of the Supplementary Materials. The EDS-derived averaged compositions were used to quantify the relative Cu/In deviations from stoichiometry across the investigated sample series.

The compositions reported in Table 1 represent averages of 5–7 EDS measurements acquired at different positions of each sample. For the representative spectra shown in the Supplementary Materials, the EDAX APEX eZAF quantification yielded overall analysis uncertainties of 8.40–9.28%. The corresponding element-specific estimated errors were approximately 3.4% for Cu and 4.7–4.8% for In. These values characterize the analytical uncertainty of the EDS quantification and should not be interpreted as point-to-point standard deviations.

### 2.3. Dielectric and Uniaxial-Pressure Measurements

Temperature-dependent dielectric measurements were performed using an automated computer-controlled system incorporating an immersion-type cryostat [28], a Measurement Computing (Norton, MA, USA) USB-TEMP-AI data-acquisition unit, and an OWON ODP3033 (OWON Technology Inc., Zhangzhou, China) programmable dc power supply. The system allowed heating and cooling rates between 0.001 and 1 K min^−1^. Temperature was monitored using a PT100/1509A platinum resistance thermometer (TDI Ltd., Southport, UK). The temperature-measurement resolution was 0.001 K, with an absolute accuracy of ±2.9 K at 73 K and ±0.27 K at 373 K.

The complex dielectric permittivity was measured using a GW Instek (Good Will Instrument Co., Ltd., New Taipei City, Taiwan) LCR-819 LCR meter over the frequency range 12 Hz–100 kHz [29]. Plate-like samples with thicknesses of approximately 50–100 µm were used, and gold electrodes with areas between approximately 2 × 2 and 5 × 5 mm^2^ were deposited on the major surfaces. Because these faces are parallel to the structural layers, the ac electric field was applied approximately normal to the layers. The ac measuring voltage was adjusted according to sample thickness so that the electric field amplitude was maintained at *E_ac_* = 1 V cm^−1^ for all samples. This field is far below the reported coercive field of *E_c_* = 77 kV cm^−1^ [11], ensuring operation within the linear dielectric response regime.

To minimize the influence of previous thermal history (“memory” effects), all crystals were held at 100 °C for 3 h before each measurement series. Each dielectric measurement was repeated at least five times under identical conditions. The run-to-run variation of the transition temperature was below 0.1 K, which is smaller than the absolute accuracy of the platinum resistance thermometer (±0.27 K at 373 K). The dielectric data were reproducible within approximately 1%. Unless otherwise stated, temperature scans used for the comparison of the different compositions were performed at a cooling rate of 0.1 K min^−1^, which minimized temperature gradients across the thin crystals and reduced the broadening of the dielectric anomaly near *T_C_*.

For measurements under uniaxial compression, the mechanical load was transmitted to the gas transport-grown CuInP_2_S_6_ crystal through a quartz rod with a diameter of 5 mm and a gold-coated quartz plate. One end of the rod was brought into contact with the sample assembly, while calibrated masses from a standard laboratory weight set were placed on the opposite end. The applied force was calculated as F = mg, and the nominal applied stress was determined as *σ* = *F*/*A_r_*, where *A_r_* = *πd*^2^/4 is the cross-sectional area of the d = 5 mm quartz rod (*A_r_* ≈ 19.6 mm^2^). The gold-coated quartz plate distributed the force over the sample surface and simultaneously served as one electrical electrode; the opposite electrode was connected to the base of the cryostat sample holder. In this geometry, the compressive stress was applied approximately normal to the structural layers. Before every pressure-measurement cycle, the sample was annealed for 3 h at 100 °C in the paraelectric phase, and each measurement was repeated at least five times. The run-to-run variation of the transition temperature was below 0.1 K.

### 2.4. Raman Spectroscopy

Room-temperature Raman spectra were acquired in reflection geometry using a Renishaw inVia (Renishaw plc, Wotton-under-Edge, UK) Raman microscope coupled to a Leica DM 2700 (Leica Microsystems GmbH, Wetzlar, Germany) optical microscope. An excitation wavelength of 785 nm was used, and the spectral resolution was approximately 0.7 cm^−1^. The recorded spectra extended from below 50 cm^−1^ to approximately 600 cm^−1^, covering both low-frequency cation/lattice modes and higher-frequency internal vibrations of the P_2_S_6_ units. For cross-sample comparison, the Raman spectra were normalized to the intensity of the prominent P–P-related Raman band. Spectral acquisition and processing were performed using Renishaw WiRE software (version 5.7).

For quantitative comparison, selected Raman bands were fitted using pseudo-Voigt profiles with a local linear background. Peak positions, full widths at half maximum (FWHM), and peak intensities normalized to the fitted P–P-related band were extracted using the same fitting procedure and spectral windows for all four samples.

### 2.5. Piezoresponse Force Microscopy

Piezoresponse force microscopy (PFM) measurements were performed under ambient conditions using a Dimension Icon atomic force microscope (Bruker, Billerica, MA, USA) equipped with Pt–Ir-coated SCM-PIT conductive probes. Both single-frequency PFM imaging and band-excitation imaging/local polarization-switching measurements were employed. Local switching measurements were typically performed using tip biases between −10 and +10 V.

Because CuInP_2_S_6_ exhibits a negative longitudinal piezoelectric coefficient *d*_33_, the PFM phase contrast was interpreted accordingly, with a 180° phase difference corresponding to antiparallel out-of-plane polarization states.

The CuInP_2_S_6_ samples were electrically connected to metal sample disks (Ted Pella, Prod. No. 16218) using conductive silver paste (Ted Pella, Prod. No. 16035), with the disks connected to ground. Fresh surfaces were prepared immediately before measurement by mechanical exfoliation using the “Scotch-tape” method [30]. PFM images were processed using Gwyddion v.2.49.20171219 [31]. The PFM response was calibrated using a periodically poled lithium niobate reference sample (AR-PPLN 900.241, Asylum Research, Santa Barbara, CA, USA).

For quantitative analysis of the local switching loops, the negative- and positive-branch switching thresholds, *V_sw_*^−^ and *V_sw_*^+^, were determined from the minima of the corresponding PFM amplitude branches; the phase loops were used to verify the associated ~180° phase reversal. The local switching half-width and imprint voltage were calculated as V_c_* = (*V_sw_*^+^ − *V_sw_*^−^)/2 and *V_imprint_* = (*V_sw_*^+^ + *V_sw_*^−^)/2, respectively. For phase-state population analysis, all four PFM phase maps were processed using the same automated procedure: the RGB images were converted to CIELAB color space, the two dominant phase-contrast populations were separated using an Otsu threshold applied to the L* channel, and pixels within a transition interval of ±10% of the separation between the two population medians were excluded as domain-boundary or ambiguous pixels. The remaining pixels were used to calculate the relative areas of the two phase-contrast states.

### 2.6. Use of Generative AI Tools

During the preparation and revision of this manuscript, ChatGPT (OpenAI, GPT-5.6) was used for language refinement, restructuring and drafting support, and to assist with the code-based quantitative post-processing of already acquired Raman and PFM datasets; no experimental image content or quantitative data were generated or altered by GenAI. ChatGPT was not used for experimental design, data acquisition, or the generation of experimental data. All analysis procedures and numerical outputs were reviewed and verified by the authors.

## 3. Results

### 3.1. Dielectric Data

**Figure 3:**
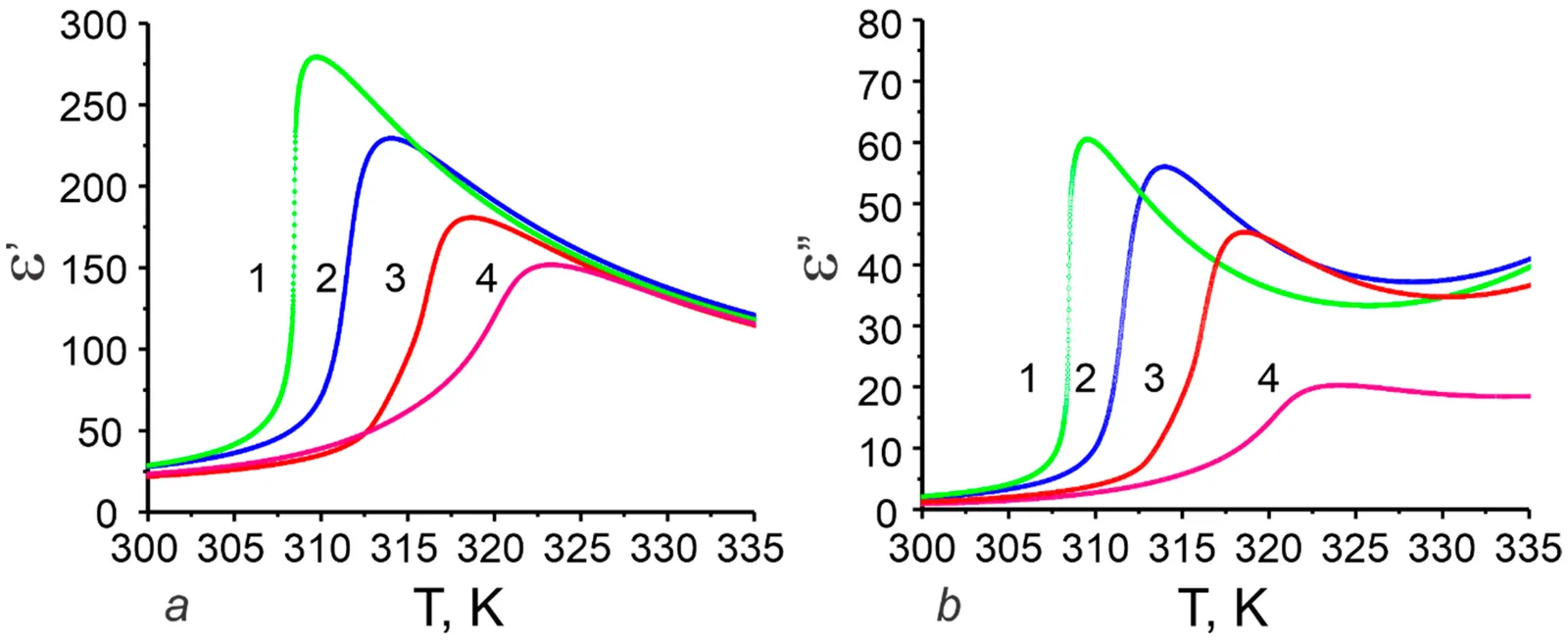
Temperature dependences of (**a**) the real ε′ and (**b**) the imaginary ε″ components of the complex dielectric permittivity at 10 kHz for CuInP_2_S_6_ crystals: 1—Bridgman–Stockbarger growth; 2—Cu-enriched CuInP_2_S_6_ + Cu_2_S; 3—gas transport growth; 4—In-enriched CuInP_2_S_6_ + In_2_S_3_. The curves numbered 1–4 are shown in green, blue, red, and magenta, respectively.

**Figure 4:**
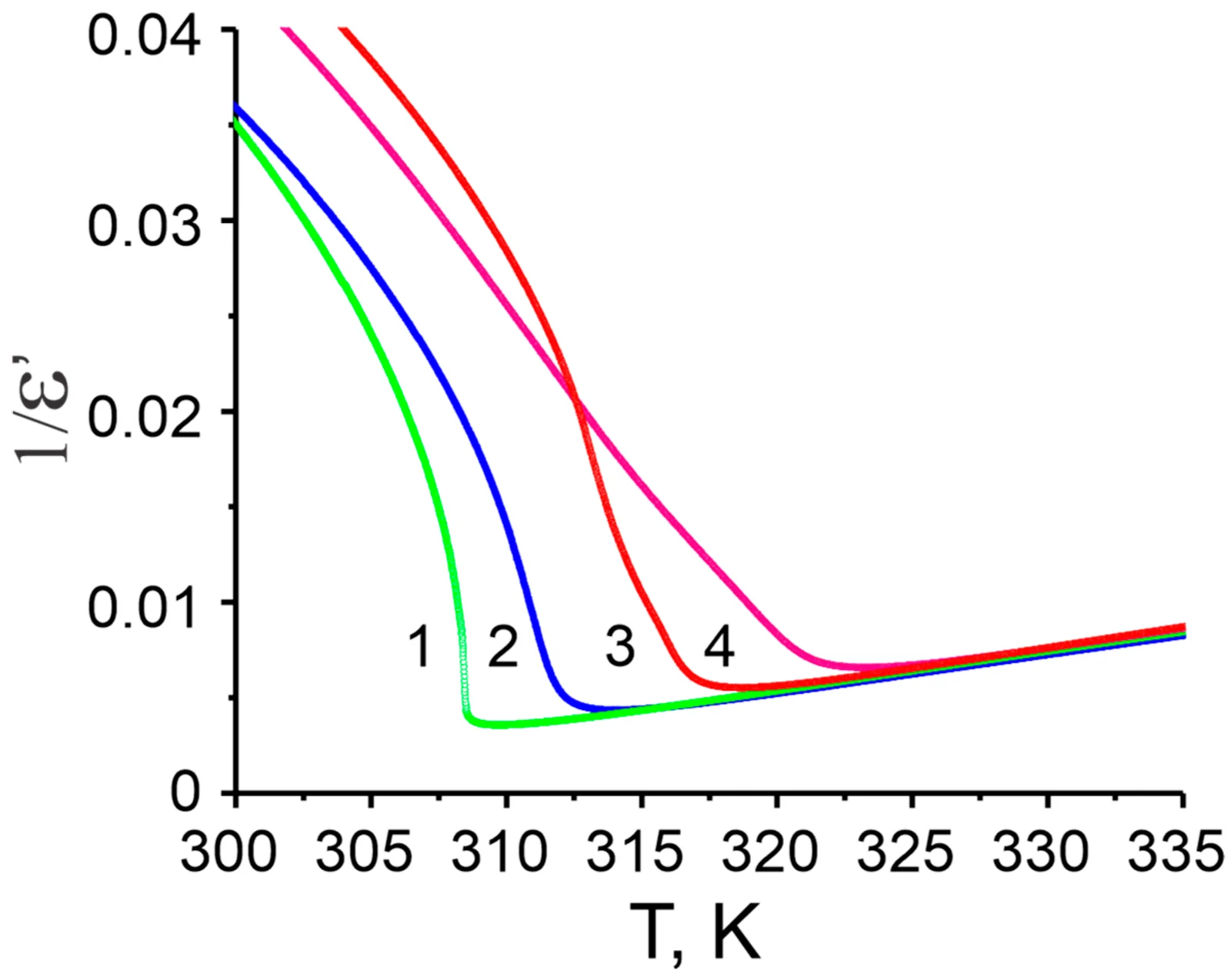
Temperature dependence of the inverse real dielectric permittivity, 1/ε′, in the phase-transition region of CuInP_2_S_6_ crystals: 1—Bridgman–Stockbarger growth; 2—Cu-enriched CuInP_2_S_6_ + Cu_2_S; 3—gas transport growth; 4—In-enriched CuInP_2_S_6_ + In_2_S_3_. The curves numbered 1–4 are shown in green, blue, red, and magenta, respectively.

**Figure 5:**
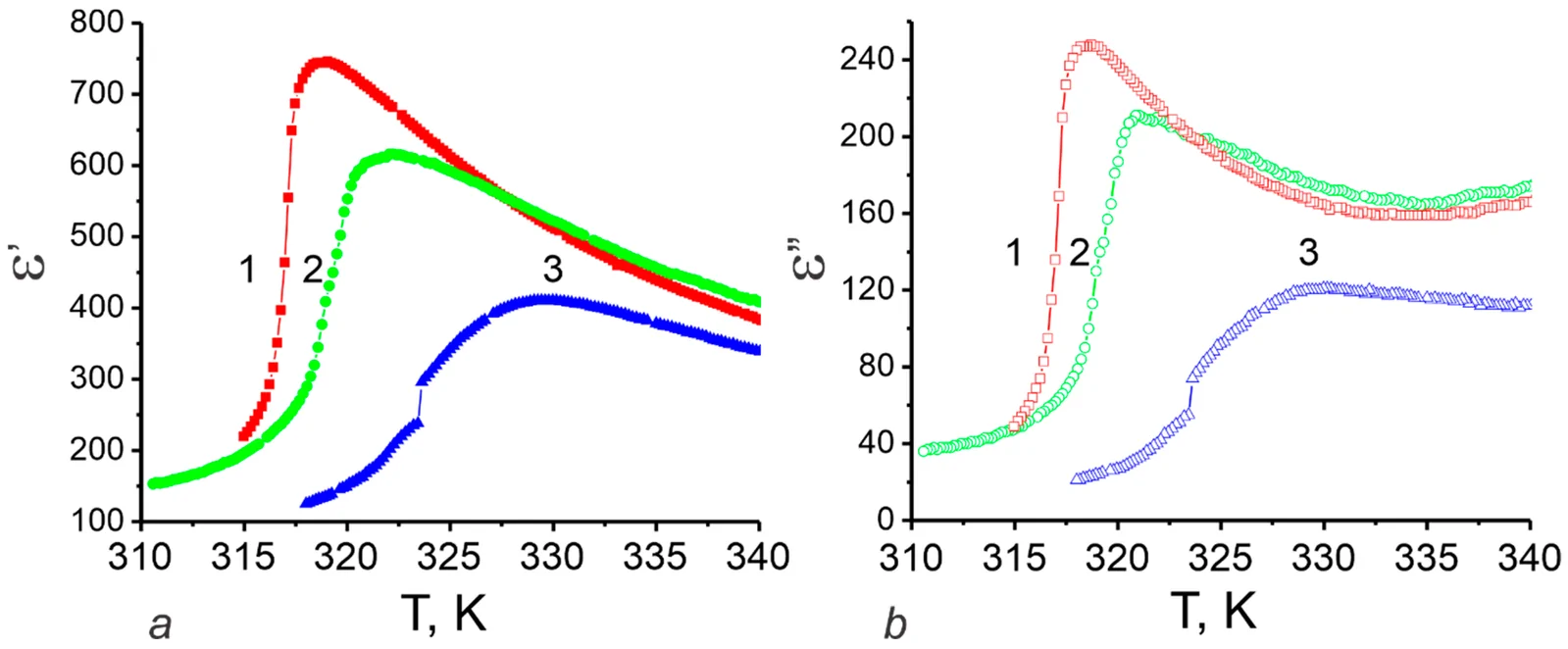
Temperature dependences of (**a**) ε′ and (**b**) ε″ for the gas transport-grown CuInP_2_S_6_ crystal under uniaxial compression: 1—0 MPa; 2—5 MPa; 3—25 MPa. Curves 1, 2, and 3 are shown in red, green, and blue, respectively.

The temperature dependences of the real and imaginary components of the complex dielectric permittivity for the four investigated CuInP_2_S_6_ crystal types are compared in Figure 3. For direct comparison, the curves in Figure 3, Figure 4 and Figure 5 were recorded at a frequency of 10 kHz. The complete temperature–frequency dependences are presented in Figures S5–S8 of the Supplementary Materials. These data show a pronounced increase in the dielectric response and losses at low frequencies, consistent with a substantial contribution from electrical conductivity and Cu-ion transport [32]. At 10 kHz, this low-frequency conductive contribution is strongly reduced, providing a more reliable comparison of the dielectric anomalies associated with the ferrielectric phase transition.

The complex dielectric permittivity is represented as

$$\varepsilon^{*}(T,f)=\varepsilon \prime (T,f)-i\varepsilon \prime \prime (T,f)$$

Using the $e^{i\omega t}$ convention, ε′ and ε″ are the real and imaginary (dielectric-loss) components, respectively. The four samples exhibit pronounced differences in both the position and magnitude of the dielectric anomaly. The transition temperatures determined from the dielectric response increase in the sequence 307 K for the Bridgman–Stockbarger crystal, 312 K for the Cu-enriched CuInP_2_S_6_ + Cu_2_S crystal, 317 K for the gas transport crystal, and 322 K for the In-enriched CuInP_2_S_6_ + In_2_S_3_ crystal (Table 1). Thus, deviations from stoichiometry are accompanied by systematic shifts of the ferrielectric transition together with substantial changes in the peak values of ε′ and ε″.

It should be emphasized that composition and growth route are not independently varied for every sample in the present set. Therefore, the observed trends should not be interpreted as arising from Cu/In stoichiometry alone. Stoichiometry provides the dominant thermodynamic contribution to the shift of *T_C_*, whereas growth-related defects can additionally modify dielectric broadening, internal fields, and domain-wall pinning.

The gas transport crystal, which has a near-stoichiometric EDS composition, is used here as a practical reference state. Relative to this sample, In enrichment shifts the transition to higher temperature and markedly reduces both ε′ and ε″. This behavior is consistent with the spontaneous chemical phase separation reported for Cu-deficient, In-rich CuInP_2_S_6_ [13,14], which produces coexisting ferrielectric CuInP_2_S_6_ (CIPS) and centrosymmetric In_4_/_3_P_2_S_6_ (IPS) chemical domains. The reduced fraction of the ferrielectric CIPS component decreases the macroscopic dielectric response, while the lattice mismatch between the CIPS and IPS regions generates compressive strain. The resulting effective positive chemical pressure stabilizes the ferrielectric state and shifts the transition toward a higher temperature [13,14].

The systematic displacement of the dielectric anomaly is also evident in the inverse permittivity 1/ε′ shown in Figure 4. The minima and the subsequent high-temperature branches shift progressively toward higher temperature in the same sequence as the transition temperatures listed in Table 1, making the composition-dependent displacement of the phase transition particularly clear.

An additional characteristic of the In-enriched sample is the substantially lower dielectric loss in the paraelectric region (Figure 3b). Cu-ion redistribution among nonequivalent crystallographic sites is closely connected with the dielectric and ionic response of CuInP_2_S_6_ [11]. The suppressed ε″ observed in the In-rich sample therefore suggests that the CIPS/IPS chemical microstructure, together with the associated strain and pinning, restricts the long-range contribution of mobile Cu-related species to the dielectric response. In contrast, Cu enrichment shifts the transition below that of the gas transport reference and retains a comparatively strong dielectric loss contribution, indicating an opposite trend in the stability and mobility of the Cu sublattice.

To establish an experimental reference for the effect of mechanical compression, the dielectric response was also measured under uniaxial pressure. As shown in Figure 5, increasing compression shifts both the ε′ and ε″ anomalies toward higher temperature. The transition temperature increases from approximately 317 K at 0 MPa to about 319 K at 5 MPa and approximately 323 K at 25 MPa. Over this low-pressure interval, the shift is approximately linear and corresponds to a pressure coefficient of the order of 2 × 10^2^ K GPa^−1^.

**Figure 6:**
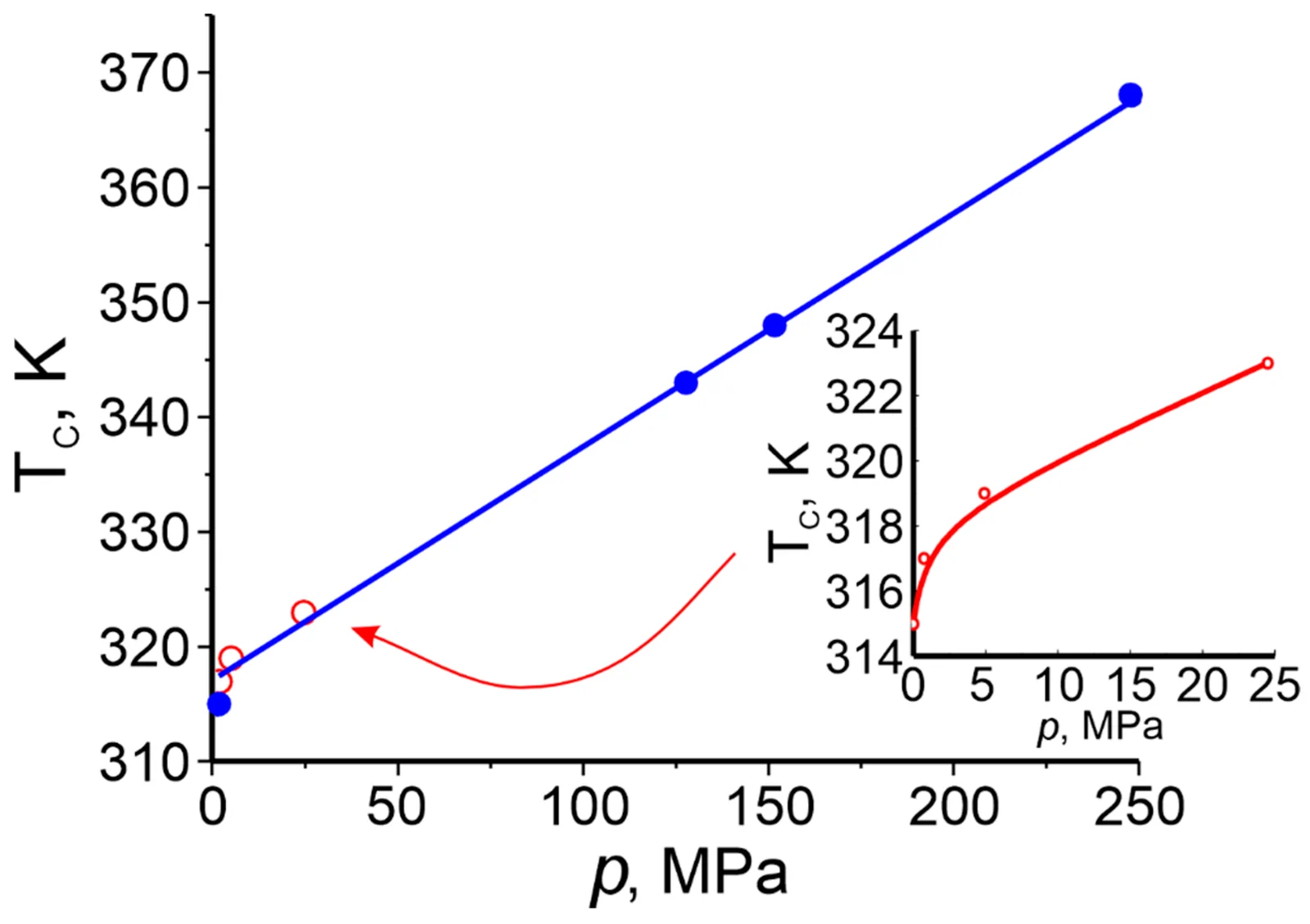
Pressure dependence of the ferrielectric transition temperature *T_C_*. Red symbols and the inset show the present uniaxial pressure data; blue symbols show data from Refs. [18,19]. The blue solid line connects the literature points as a guide to the eye, and the curved arrow indicates the enlarged low-pressure region shown in the inset.

Figure 6 compares the present low-pressure results with previously reported pressure dependences [18,19]. Both datasets show a positive pressure coefficient of the transition temperature. The agreement demonstrates that relatively modest mechanical compression stabilizes the ferrielectric phase and provides a direct experimental reference for the comparison between mechanical pressure and stoichiometry-induced internal strain developed in Section 3.2.

### 3.2. Comparison of Stoichiometry and Pressure Effects

The dielectric results reveal a direct parallel between the effects of external compression and deviations from stoichiometry. Mechanical compression shifts the ferrielectric transition toward higher temperature, while among the compositionally modified samples the same trend is observed for In enrichment. Cu enrichment produces the opposite response and shifts the transition toward lower temperature. These observations indicate that stoichiometry and external pressure act, at least in the low-pressure regime, on closely related structural degrees of freedom that control the stability of the ferrielectric state.

Previous pressure studies provide an experimental basis for comparing the hydrostatic and uniaxial responses of CuInP_2_S_6_. For uniaxial stress applied approximately along [001], the reported coefficient is *dT_C_*/*dσ*_3_ ≈ 20 K kbar^−1^ (≈200 K GPa^−1^), whereas hydrostatic pressure measurements yield *dT_C_*/*dp* ≈ 210 K GPa^−1^ [18,19,20]. The present low-pressure uniaxial measurements give a coefficient of the same order. Thus, the additional pressure scale used below is based on the experimentally established near-equality of the transition temperature pressure coefficients, rather than on an assumption that hydrostatic and uniaxial loading are mechanically equivalent.

This behavior is consistent with the order–disorder character of the transition in CuInP_2_S_6_, in which compression modifies the relative stability and occupation of nonequivalent Cu positions. At low pressures, compression is also accompanied by an enhancement of the spontaneous polarization and redistribution of Cu ions among available sites [21]. The present comparison is therefore restricted to this low-pressure regime; the more complex structural transformations reported at substantially higher pressures [24] are outside the chemical pressure analogy considered here.

For a phenomenological comparison, the stoichiometry-induced shift of the transition temperature can also be expressed as an equivalent pressure using the experimentally established low-pressure coefficient:

$$p_{eff}\;\approx \;\frac{\left[T_{C}(x)\;-\;T_{C,ref}\right]}{{\left(\partial T_{C}/\partial p\right)}_{mech}}$$

where *T_C,ref_* is the transition temperature of the near-stoichiometric reference crystal and (*∂T_C_/∂p*)*_mech_* is the mechanical pressure coefficient. This relation provides only an operational, order-of-magnitude scale for comparing the magnitude of the stoichiometry-induced *T_C_* shift with that produced by external mechanical compression. It is not intended to imply strict thermodynamic equivalence between chemical substitution and applied stress, because stoichiometric variation simultaneously alters local chemistry, defect density and (in the In-rich limit) the CIPS/IPS phase fraction.

The microscopic basis of this correspondence is particularly clear for In-rich crystals. Cu-deficient, In-enriched CuInP_2_S_6_ undergoes chemical phase separation into ferrielectric CIPS and centrosymmetric In_4/3_P_2_S_6_ (IPS) regions [13]. The lattice mismatch between these chemical domains produces strain in the CIPS component [14], so that In enrichment acts as an effective positive chemical pressure. The resulting increase in *T_C_* therefore follows the same direction as that produced by external compression.

Cu enrichment leads to the opposite tendency. The composition dependence of the interlayer spacing reported for the CIPS/IPS system [14] indicates that increasing the Cu content expands the van der Waals-separated structure relative to the In-rich limit. Accordingly, the decrease in *T_C_* observed for Cu-rich crystals can be described as a negative-pressure-like, or tensile, response. This terminology should be understood as an effective structural analogy: changes in stoichiometry simultaneously modify local chemistry, defect concentration, phase fraction, and Cu-ion configuration, and therefore cannot be reduced to mechanical pressure alone.

**Figure 7:**
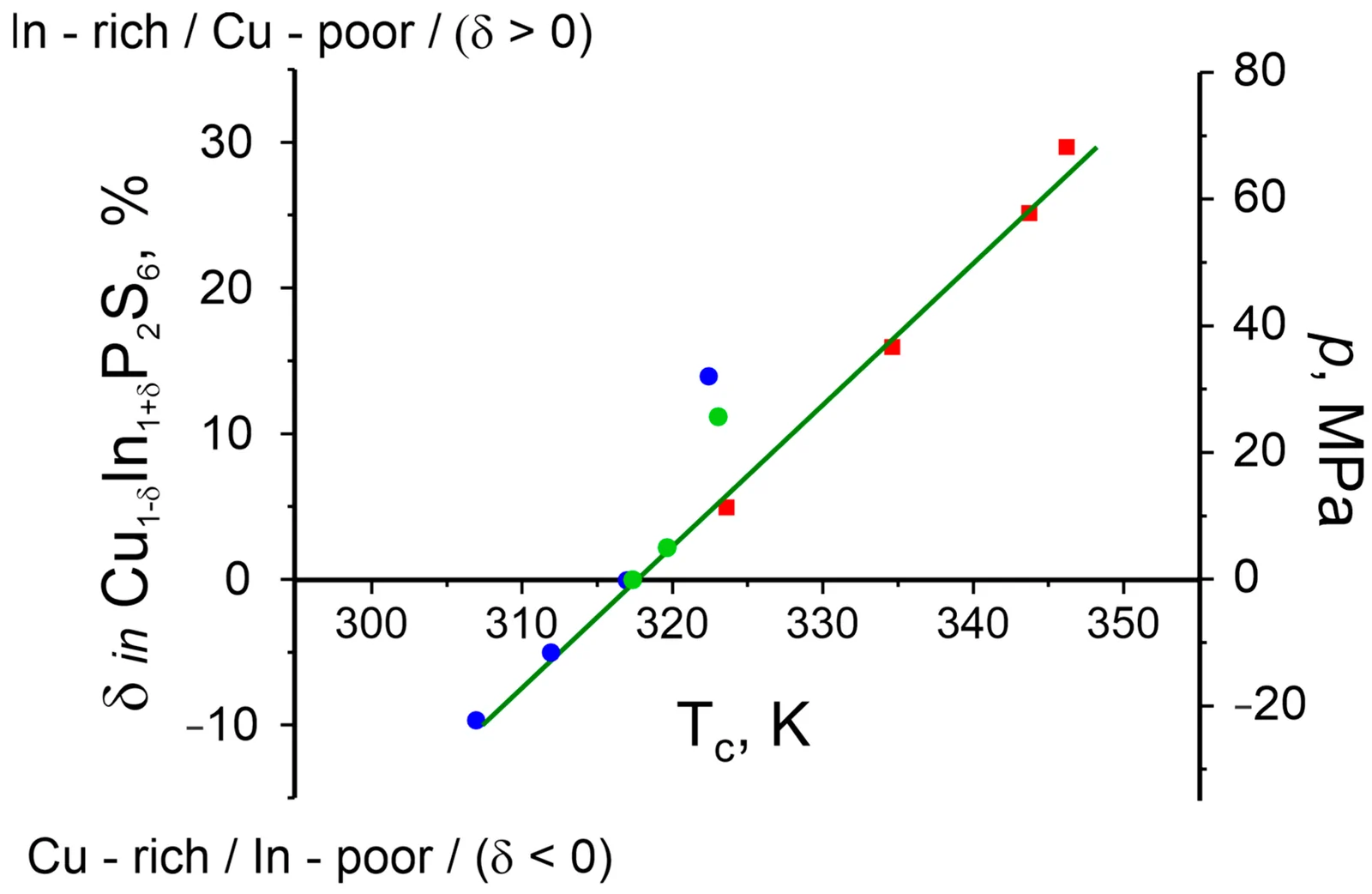
Pressure–stoichiometry–transition temperature correspondence for CuInP_2_S_6_. Blue circles represent the current experimental data on stoichiometric deviations, with positive δ values corresponding to In enrichment and negative δ values to Cu enrichment. Green circles represent the present uniaxial pressure data, plotted using the equivalent-pressure scale on the right-hand axis. Red squares show literature composition-dependent data from Ref. [13], with transition temperatures shifted by +9.4 K to align the Cu_0.99_In_1.05_P_2.04_S_6_ reference composition (*T_C_* = 307.6 K) with the transition temperature of the present gas transport-grown reference crystal (*T_C_* = 317 K). The solid green line illustrates the phenomenological correspondence between stoichiometric deviations and equivalent mechanical pressure. This comparison does not imply strict thermodynamic equivalence between chemical and externally applied pressure.

Figure 7 thus summarizes an experimentally useful correspondence between stoichiometry and pressure. Both parameters influence Cu-site occupancy (Cu1, Cu2, and Cu3) [11], interlayer geometry, and the barriers governing Cu-ion redistribution, thereby shifting the balance between long-range ferrielectric order and local dynamic disorder. This framework provides the basis for comparing the vibrational response of the same samples in Section 3.3 and, subsequently, their domain structures.

### 3.3. Raman Spectroscopy Results

**Figure 8:**
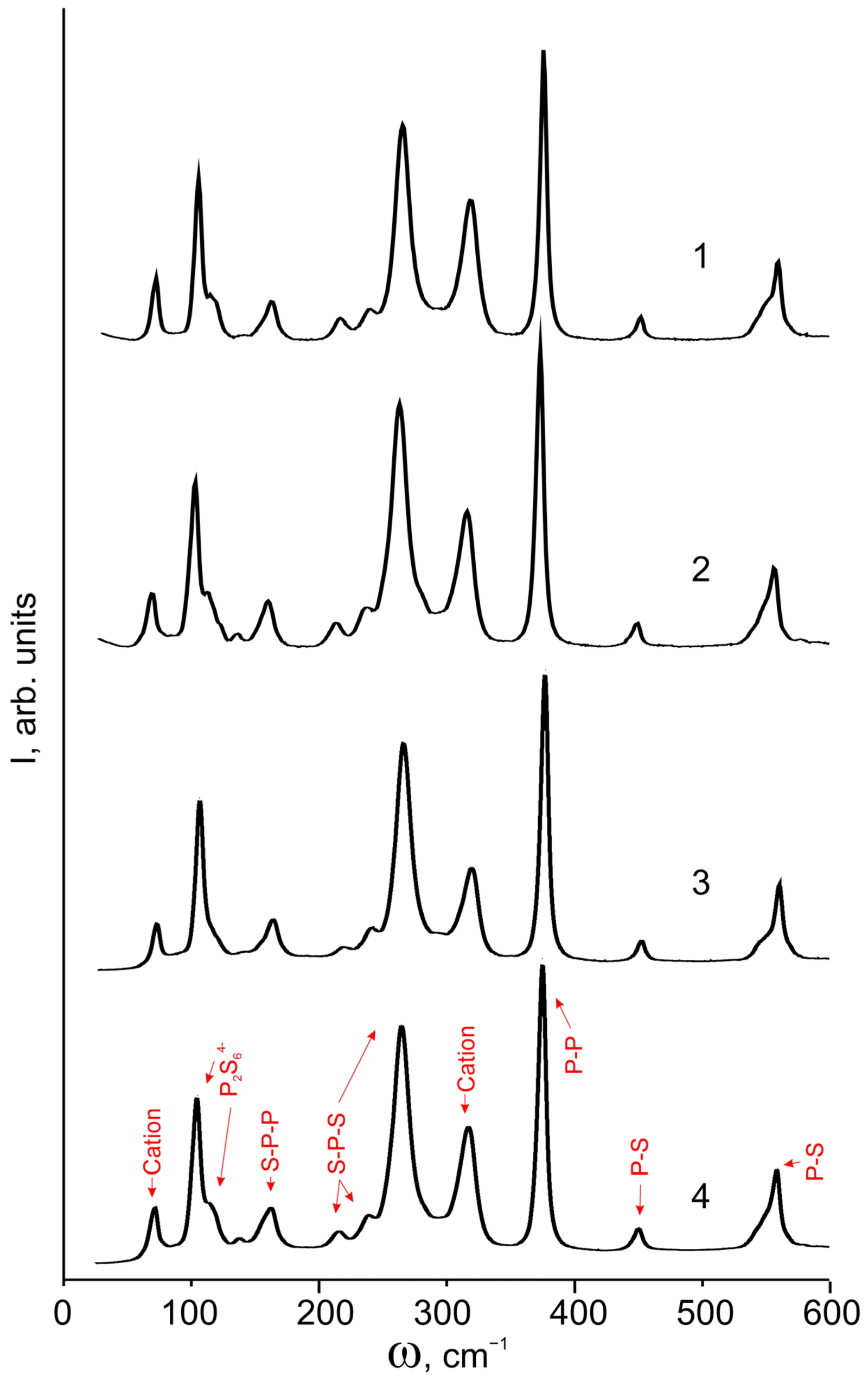
Room-temperature Raman spectra of CuInP_2_S_6_ crystals: 1—Cu-enriched CuInP_2_S_6_ + Cu_2_S; 2—In-enriched CuInP_2_S_6_ + In_2_S_3_; 3—Bridgman–Stockbarger growth; 4—gas transport growth. Spectra were normalized to the P–P-related band and vertically offset for clarity; mode assignments for sample 4 follow Ref. [23].

Room-temperature Raman spectra of CuInP_2_S_6_ crystals grown by different methods and with controlled deviations from stoichiometry are compared in Figure 8. The spectra extend from below 50 cm^−1^ to approximately 600 cm^−1^, encompassing both low-frequency lattice and cation-related modes and higher-frequency internal vibrations of the P_2_S_6_ units. For the comparison of relative spectral intensities, all spectra were normalized to the intensity of the prominent P–P-related Raman band and were subsequently vertically offset for clarity. All four samples retain the characteristic vibrational fingerprint of CuInP_2_S_6_ reported previously [23], indicating that the principal structural framework is preserved. At the same time, reproducible differences in the relative intensities, linewidths, and positions of several bands demonstrate that the lattice dynamics are sensitive to both growth conditions and cation stoichiometry.

According to the mode assignments established in Ref. [23], the low-frequency part of the spectrum is dominated by lattice and framework vibrations involving Cu- and In-related displacements together with deformations of the sulfur coordination polyhedra, whereas the higher-frequency region contains predominantly internal vibrations of the P_2_S_6_ units. The spectra below approximately 200 cm^−1^ contain several well-resolved features whose relative intensities, widths, and positions differ among the four samples. Because these modes involve the cation sublattices and relatively soft framework deformations, their sensitivity to composition and growth conditions provides direct evidence that the local cation environment and the low-energy lattice dynamics are modified by stoichiometry.

Clear composition-dependent differences are also observed in the intermediate-frequency region. In particular, the relative intensities, positions, and mode-dependent linewidths of the bands in the approximately 250–350 cm^−1^ range vary among the samples. The In-enriched crystal shows a pronounced redistribution of spectral weight in this region together with small frequency shifts, consistent with the chemically heterogeneous CIPS/IPS state and its associated local strain and modified cation–framework coupling. The Cu-enriched crystal exhibits a different spectral profile, indicating that excess Cu modifies the local potential landscape and cation–framework interactions in a distinct manner.

At higher frequencies, the strong bands associated predominantly with internal vibrations of the P_2_S_6_ structural units remain present in all four samples. The prominent P–P-related band near 375 cm^−1^ and the P–S-related features in the approximately 450–600 cm^−1^ region [23,24] therefore confirm that the basic anionic framework remains intact. Because the spectra were normalized to the P–P-related band, the observed changes in the other modes represent a redistribution of Raman intensity relative to this internal structural reference. The comparatively stronger changes in the low- and intermediate-frequency regions indicate that stoichiometry primarily perturbs the softer cation- and framework-related degrees of freedom, rather than reconstructing the P_2_S_6_ units.

Pressure-dependent Raman studies at substantially higher, multi-GPa pressures reveal a complex and non-monotonic evolution of the CIPS vibrational spectrum [33]. Those pressure regimes lie far outside the low-pressure range relevant to the present pressure–stoichiometry comparison, and the Raman spectra are therefore not used here to assign a quantitative equivalent mechanical pressure to a particular composition. Instead, they provide an independent probe showing that stoichiometric modification affects the low-energy cation–lattice degrees of freedom that are central to the ferrielectric instability.

Quantitative fitting confirms the composition-dependent changes visible in Figure 8. Relative to the gas transport reference, small but systematic frequency shifts are observed in the compositionally modified crystals. The P–P-related band shifts from 374.69 cm^−1^ in the gas transport crystal to 374.01 cm^−1^ in the Cu-enriched sample and 373.67 cm^−1^ in the In-enriched sample. A similar trend is observed for the high-frequency band near 558 cm^−1^, which shifts from 557.68 cm^−1^ to 557.09 and 556.30 cm^−1^, respectively. Pronounced changes also occur in relative spectral weight: the band near 317 cm^−1^ increases from I/I_P–P_ = 0.364 in the gas transport crystal to 0.439 in the Cu-enriched sample, while the In-enriched sample exhibits the strongest relative intensity of the band near 264 cm^−1^ (I/I_P–P_ = 0.789, compared with 0.729 for the gas transport crystal). Linewidth changes are mode-dependent rather than uniform; for example, the band near 558 cm^−1^ broadens from 8.09 cm^−1^ in the gas transport crystal to 9.43 cm^−1^ in the In-enriched sample. The complete quantitative comparison of peak positions, normalized intensities, and FWHM values is provided in Table S1 of the Supplementary Materials.

Taken together, the Raman data provide an independent microscopic link between stoichiometry and the Cu-ion/lattice dynamics of CIPS. The composition-dependent shift of the ferrielectric transition is accompanied by systematic modifications of low-energy modes associated with cation displacement and framework deformation, while the internal P_2_S_6_ vibrational framework remains largely preserved. Combined with the dielectric and theoretical results, these observations support a common microscopic picture in which stoichiometry modifies the Cu-ion configurational landscape, local strain, and interlayer coupling. The pressure analogy remains phenomenological: Raman spectroscopy identifies changes in the relevant lattice degrees of freedom, but does not by itself establish a quantitative pressure equivalence.

## 4. Microscopic Interpretation and First-Principles Support

### 4.1. Cu-Ion Energy Landscape and the Stoichiometry–Pressure Correspondence

The experimental results presented above can be understood within a common microscopic picture in which the ferrielectric state of CuInP_2_S_6_ is governed by the occupation and redistribution of Cu ions among several nonequivalent positions, together with the counter-displacement of the In sublattice. Electron-diffraction measurements show that the probability distribution of Cu becomes progressively more symmetric on heating through the ferrielectric transition, while a small but finite population of interlayer tetrahedral Cu sites appears at higher temperatures [11]. Thus, the transition is intrinsically connected with a multi-site, multiwell Cu-ion energy landscape rather than with a single displacive coordinate.

Microscopic descriptions based on local multiwell potentials and coupled cation–lattice degrees of freedom have been developed for thiophosphate ferroelectrics [34,35] and, more specifically, for CIPS within a mixed Ising-type description [36]. These approaches emphasize that relatively small changes in local bonding, interlayer coupling, or site energies can redistribute the statistical occupation of competing Cu positions. Pressure studies of related thiophosphates likewise demonstrate that the polar state is highly sensitive to changes in bond character and lattice geometry [19,37]. For the present work, however, the experimentally relevant variables are the Cu-site energy landscape and interlayer coupling, because both are directly affected by external stress and by composition-dependent internal strain.

A particularly useful phenomenological framework was developed by Morozovska et al. [38] using an eighth-order Landau–Ginzburg–Devonshire (LGD) expansion for CIPS. Importantly, the pressure-dependent dielectric data used to parameterize this model included the earlier measurements of compositionally modified and uniaxially compressed CuInP_2_S_6_ crystals reported in our FM&NT-2020 work [39]. In the homogeneous, zero-field limit, the stress-dependent part of the reconstructed free-energy density can be written in the form

$$g_{LGD}=\left[\frac{\alpha_{T}(T-T_{C})}{2}-\sigma_{i}Q_{i3}\right]P_{3}^{2}+\left(\frac{\beta }{4}-\sigma_{i}Z_{i33}\right)P_{3}^{4}+\frac{\gamma }{6}P_{3}^{6}+\frac{\delta }{8}P_{3}^{8}$$

where *P*_3_ is the polarization component normal to the layers, *σ_i_* denotes the applied stress components (Voigt notation), and *Q_i_*_3_ and *Z_i_*_33_ are the linear and nonlinear electrostriction coefficients, respectively [38]. The inclusion of the eighth-order term is essential for reproducing the multiwell thermodynamic potential and the experimentally observed pressure dependence of CIPS. Both sets of coefficients are strongly temperature-dependent and possess unconventional (partly inverted) signs that are characteristic of CIPS (see Supplementary Materials Section S3). In particular, the longitudinal linear coefficient *Q*_33_ is negative over a wide temperature range, which accounts for the experimentally observed negative piezoelectric coefficient *d*_33_ of bulk CIPS. The nonlinear coefficients *Z_i_*_33_ are essential for reproducing the multiwell thermodynamic potential and the experimentally observed pressure dependence of the transition temperature. Their inclusion allows intermediate negative (tensile) pressure to render the competing potential wells shallow and nearly equal in energy, producing an almost flat free-energy profile. Such a flattened multiwell landscape is fully consistent with the experimental signatures observed for Cu enrichment: reduced *T_C_*, enhanced dielectric loss, Raman evidence of modified low-frequency cation–lattice dynamics, and the fragmented, weakly correlated domain state revealed by PFM. In this regime the energetic barriers between competing Cu-site configurations are lowered, increasing the configurational freedom of Cu ions and reducing the energetic preference for long-range ferrielectric order. Conversely, the response of the In-enriched crystals maps onto the positive-pressure side of the same landscape. Lattice mismatch within the CIPS/IPS chemical heterostructure generates compressive strain in the CIPS regions, deepening the polar wells, raising *T_C_* and favoring a more rigid, strongly biased domain configuration. Thus, the entire experimental series—from Cu-enriched (negative-pressure-like) through near-stoichiometric gas transport to In-enriched (positive-pressure-like)—can be placed on a single continuous axis of the multiwell LGD potential controlled by the effective interlayer strain and Cu-site energetics. The correspondence remains phenomenological rather than strictly thermodynamic, because stoichiometric variation simultaneously alters local chemistry, defect density and, in the In-rich limit, the CIPS/IPS phase fraction. Nevertheless, both chemical and mechanical control parameters act on the same microscopic degrees of freedom.

### 4.2. First-Principles Calculations

To examine how weakened interlayer coupling affects the local electronic and vibrational structure, first-principles calculations were performed for bulk CIPS and for a single-layer (1L) periodic-supercell model. The calculations were based on density functional theory [40,41] as implemented in CASTEP [42]. The exchange–correlation energy was described within the generalized-gradient approximation using the PBE functional [43], and a dispersion correction was included to account for long-range van der Waals interactions [44]. Ultrasoft pseudopotentials were employed [45]. Structural optimization was carried out before property calculations, using a plane-wave cutoff energy of 880 eV and a total-energy convergence criterion of 10^−6^ eV atom^−1^. Brillouin-zone integrations used Monkhorst–Pack k-point sampling [46], and vibrational properties were calculated within the finite-displacement approach [47].

The 1L model was generated from the bulk structural unit by introducing a 5 Å vacuum region normal to the natural layers and applying periodic boundary conditions. Because the vacuum-thickness convergence was not examined systematically, this model should be regarded as a qualitative representation of strongly reduced interlayer coupling rather than as a fully isolated monolayer. Its purpose is to identify trends in charge redistribution and lattice dynamics associated with weakening the interlayer environment, rather than to reproduce quantitatively the properties of an isolated CIPS monolayer.

### 4.3. Bulk–Single-Layer Comparison and Implications for Interlayer Coupling

The Mulliken analysis in Table 2 shows only moderate changes ongoing from the bulk structure to the 1L model, but the direction of the change is systematic; the positive charge associated with Cu and In decreases slightly, while the magnitude of the negative S charge is also reduced. The calculated values therefore indicate a modest redistribution of charge when the interlayer environment is strongly reduced, consistent with the mixed ionic–covalent character of bonding in CIPS.

**Figure 9:**
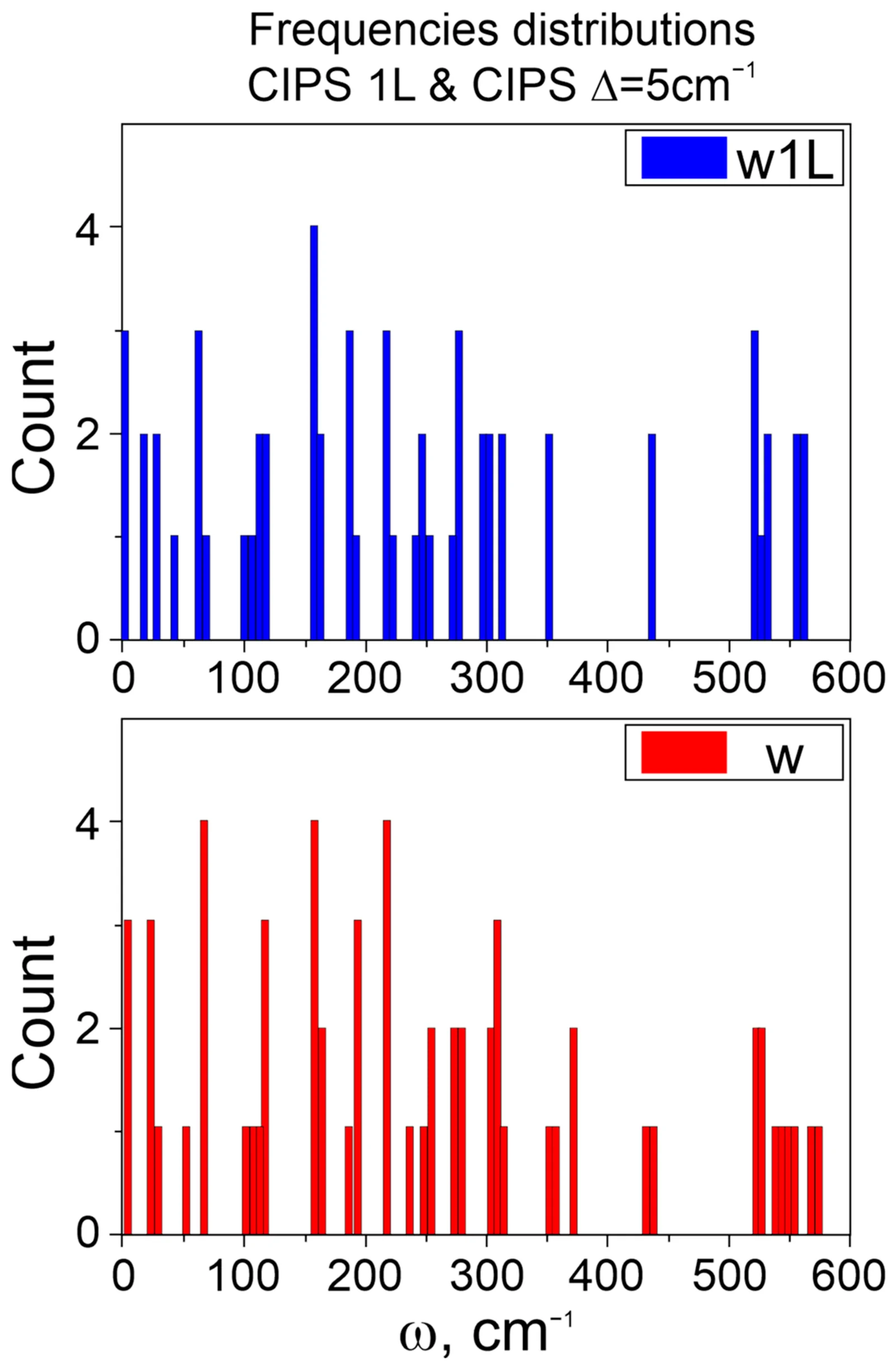
Calculated Γ-point vibrational frequency distributions for single-layer (1L, (**top**)) and bulk (**bottom**) CuInP_2_S_6_. The histogram bin width is Δ = 5 cm^−1^. In the plotted legends, “w1L” and “W” denote the 1L and bulk calculations, respectively.

The vibrational frequency distributions provide a clearer fingerprint of the loss of interlayer coupling (Figure 9). Several frequency intervals become narrower in the 1L calculation and individual modes shift, reflecting the strongly reduced interlayer coupling and the relaxation of the 1L periodic model. The changes are not consistent with a simple uniform expansion of all bonds: for example, the calculated monolayer thickness changes from 3.382 to 3.397 Å and the layer thickness parameter from 9.922 to 9.941 Å, whereas the P–P distance remains essentially unchanged (2.250 and 2.249 Å for bulk and 1L, respectively). The calculated response is therefore better described as a redistribution of interatomic interactions caused by the modified interlayer boundary condition.

**Figure 10:**
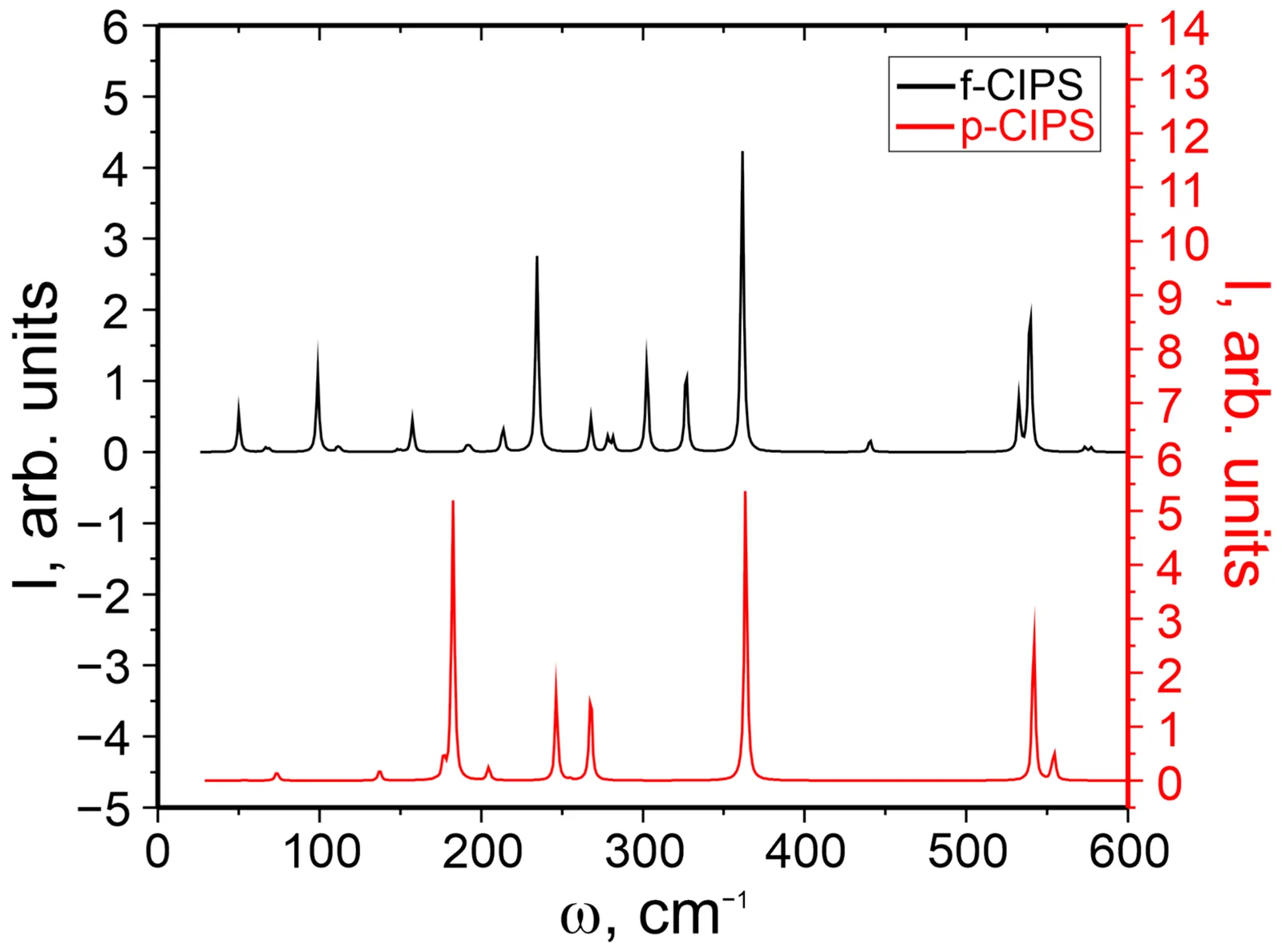
Calculated Raman spectra of bulk CuInP_2_S_6_ in the ferrielectric (f-CIPS) and paraelectric (p-CIPS) structures. The two spectra are shown using separate intensity axes for clarity; the horizontal axis represents Raman shift in cm^−1^.

The sensitivity of the lattice dynamics to the polar state is independently illustrated by the calculated Raman spectra of bulk CIPS (Figure 10). The ferrielectric and paraelectric structures show pronounced differences in the number, positions, and relative intensities of Raman-active features, confirming that the vibrational spectrum is strongly coupled to the Cu-ion configuration and the associated structural relaxation. This result is consistent with the experimental Raman trends discussed in Section 3.3, where composition-dependent changes are particularly evident in the low-frequency cation/lattice modes and in the intermediate-frequency region involving coupled cation–framework motions.

Overall, the LGD and first-principles results support the same physical picture from complementary scales. The phenomenological model [38] shows that negative pressure can flatten the multiwell free-energy landscape, while the 1L calculations demonstrate that weakening the interlayer environment measurably modifies charge redistribution and lattice dynamics. The 1L model should therefore be viewed as a qualitative limiting case of reduced interlayer coupling rather than as a literal representation of either an isolated monolayer or Cu-rich CIPS. Within this interpretation, Cu enrichment moves CIPS toward a softer, negative-pressure-like state, whereas In-rich CIPS/IPS heterostructures generate an internal compressive environment closer to the positive-pressure side. This microscopic framework provides a direct link between stoichiometry, lattice dynamics, and the strongly composition-dependent ferrielectric domain structures discussed in the following section.

## 5. Stoichiometry- and Growth-Induced Evolution of Domain Structure in CuInP_2_S_6_

**Figure 11:**
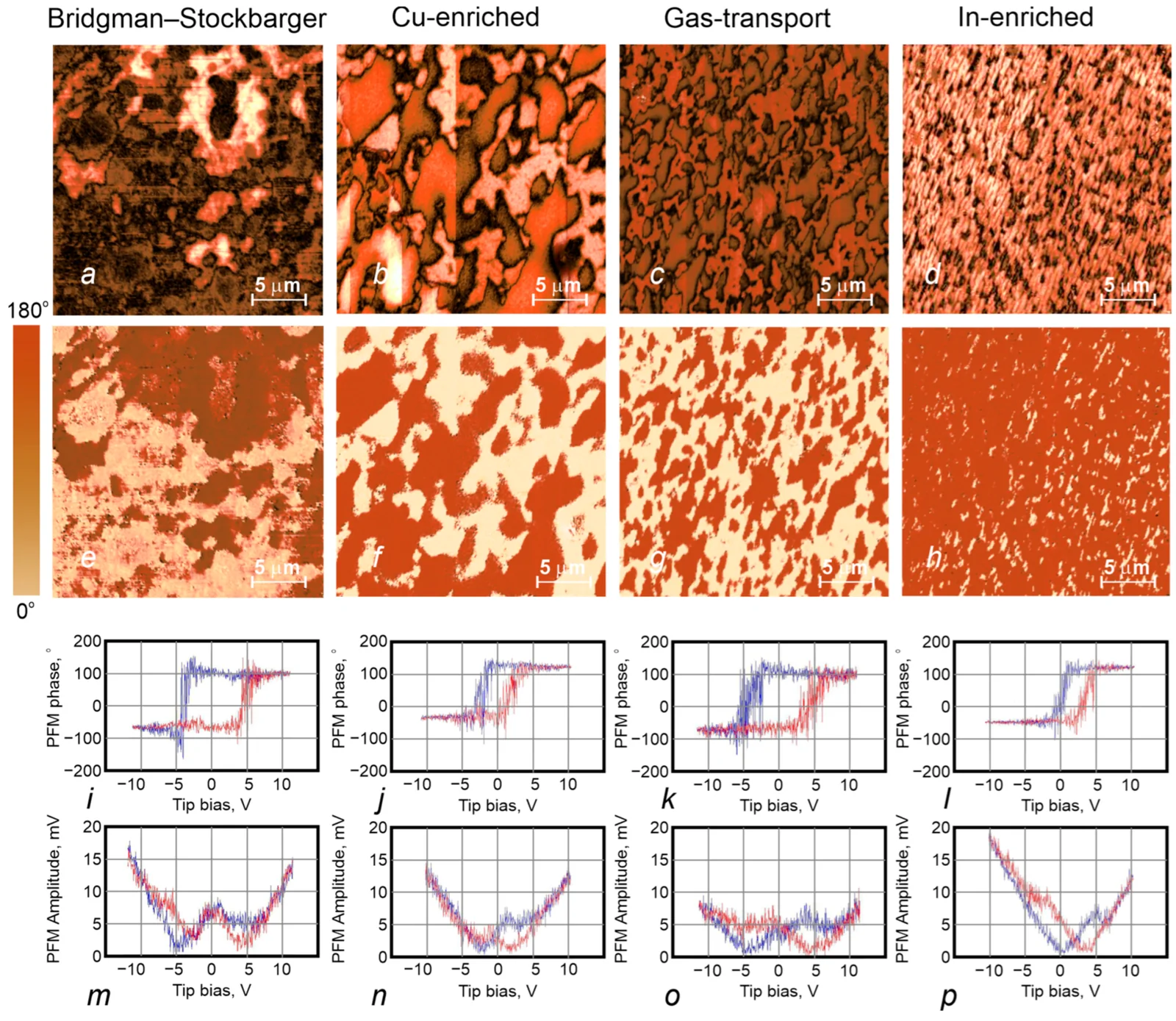
PFM amplitude (**top row**) and phase (**middle row**) images, together with local phase and amplitude switching loops (**bottom rows**), for CuInP_2_S_6_ single crystals: (**a**,**e**,**i**,**m**) Bridgman–Stockbarger growth; (**b**,**f**,**j**,**n**) Cu-enriched CuInP_2_S_6_ + Cu_2_S; (**c**,**g**,**k**,**o**) gas transport growth; and (**d**,**h**,**l**,**p**) In-enriched CuInP_2_S_6_ + In_2_S_3_. The phase images show pronounced differences in domain morphology, from large irregular domains in the melt-grown crystal to a comparatively uniform fine-domain pattern in the gas transport sample and a strongly biased domain population in the In-enriched crystal. Scale bars: 5 µm. The red and blue traces in the local switching plots distinguish the two opposite voltage-sweep branches.

The pressure–stoichiometry correspondence established from the dielectric and theoretical analyses, together with the Raman evidence for composition-dependent changes in lattice dynamics, is also reflected at the mesoscale in the polar domain structure. Figure 11 compares PFM amplitude and phase images with local switching loops for CuInP_2_S_6_ crystals grown by the Bridgman–Stockbarger and gas transport methods and for crystals prepared from Cu_2_S- and In_2_S_3_-enriched starting compositions. All four samples exhibit an approximately 180° PFM phase contrast, consistent with antiparallel out-of-plane polarization states, but the domain size, spatial uniformity, and local switching behavior differ markedly. These differences show that both stoichiometry and the defect landscape introduced during crystal growth strongly influence the organization of the ferrielectric state.

### 5.1. Reference Domain State and Growth-Induced Defect Pinning

Among the investigated samples, the gas transport crystal (Figure 11c,g,k,o) provides the closest practical reference to a near-stoichiometric, weakly defect-pinned state. Its phase image consists of a dense and relatively uniform pattern of well-defined domains with sharp contrast, similar to previously reported PFM observations of CIPS [48]. The local phase loop is comparatively symmetric and exhibits only a small imprint. This behavior is consistent with a lower density of extended growth defects and internal bias fields than in crystals grown from the melt, so that the observed domain morphology is governed more strongly by the intrinsic order–disorder character of CIPS.

The Bridgman–Stockbarger crystal (Figure 11a,e,i,m) shows a distinctly different mesoscale organization. Large, irregular domains coexist with strongly nonuniform PFM amplitude contrast, while the local phase loop remains well developed. This combination suggests that the ferrielectric state itself is robust, but that domain-wall positions are strongly influenced by extended structural inhomogeneities created during melt growth. The local switching loop probes only a nanoscale region beneath the tip; consequently, a locally symmetric loop can coexist with pronounced large-scale pinning visible in the domain image.

**Figure 12:**
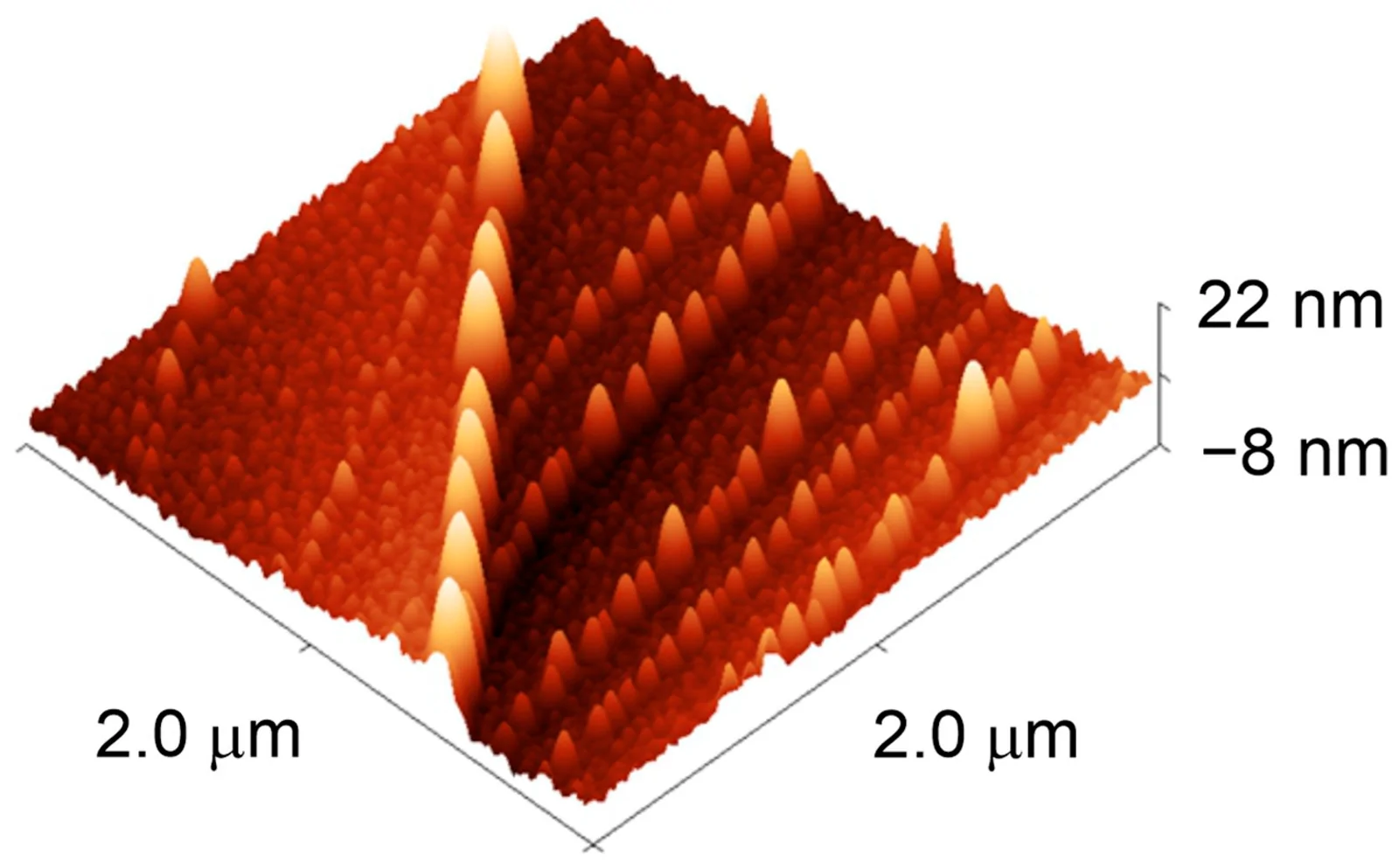
Three-dimensional AFM topography of a Bridgman–Stockbarger-grown CuInP_2_S_6_ crystal showing a discontinuity of the upper surface layer and differently oriented elongated features in the underlying region. The weak interlayer coupling allows growth-related structural inhomogeneities to vary between adjacent layers, producing a spatially heterogeneous pinning landscape. The color gradient represents the surface height, with lighter colors corresponding to higher regions and darker colors to lower regions.

The AFM topography in Figure 12 provides direct evidence for such structural heterogeneity. A discontinuity of the upper surface layer exposes an underlying region containing elongated topographic features with a different orientation. Because adjacent CIPS layers are coupled by comparatively weak van der Waals interactions, growth features and defect lines need not remain perfectly registered from one layer to the next. The resulting three-dimensional strain and defect landscape can therefore act as a network of preferential pinning sites for domain walls, explaining the large but irregular domains observed in the Bridgman–Stockbarger sample.

It should be noted that the Bridgman–Stockbarger crystal is simultaneously Cu-rich according to EDS (Table 1) and contains extended growth-induced structural inhomogeneities. The lowered transition temperature (307 K) is therefore consistent with a negative-pressure-like stoichiometric contribution, while the large, irregular domains and strong spatial nonuniformity of the PFM amplitude reflect additional domain-wall pinning by the three-dimensional defect landscape created during melt growth. These two contributions—the thermodynamic softening of the Cu-ion energy landscape and defect-mediated pinning—act concurrently and cannot be fully separated in the present data.

### 5.2. Stoichiometry-Driven Evolution of Domain Morphology and Switching

Cu enrichment produces a different type of domain disorder (Figure 11b,f,j,n). The phase pattern becomes more spatially heterogeneous, with irregular and locally discontinuous domain boundaries. Quantitatively, however, the local switching loop remains nearly centered (*V_imprint_* ≈ −0.2 V) while the local switching half-width decreases markedly to approximately 2.0 V, compared with approximately 4.6 V for the gas transport and Bridgman–Stockbarger crystals. This reduction in the characteristic local switching bias is consistent with a softer, negative-pressure-like Cu-ion energy landscape. The reduction in *T_C_* and the smaller switching half-width indicate a lower energetic separation between competing local polar configurations, while the fragmented domain morphology reflects the increased sensitivity of the polar state to local Cu-ion reconfiguration and random internal fields.

The Cu-enriched CIPS crystals offer a potentially useful route toward reducing the operating voltage of ferrielectric devices. In particular, the substantially smaller local switching half-width (approximately 2.0 V compared with 4.6 V for the near-stoichiometric reference) indicates that Cu enrichment can facilitate polarization reversal, which may be advantageous for low-voltage non-volatile memory and neuromorphic devices. However, this benefit is accompanied by increased dielectric losses, a more fragmented domain structure, and a reduction in the transition temperature to 312 K. These effects may adversely influence leakage currents, polarization retention, and the temperature range of stable device operation. Therefore, Cu enrichment should be considered a means of balancing switching accessibility against polar-state stability rather than an unconditionally beneficial modification. The present results identify this trade-off at the materials level, while direct measurements of device retention, endurance, and switching energy will be necessary to establish the optimal composition for practical applications.

The In-enriched sample (Figure 11d,h,l,p) exhibits the opposite limiting behavior. The phase image is dominated by one polarization orientation, with only sparse regions of opposite contrast, and the local phase loop shows a pronounced bias.

The quantitative PFM analysis reinforces this interpretation. The In-enriched sample exhibits *V_sw_*^−^ ≈ +0.1 V and *V_sw_*^+^ ≈ +3.5 V, corresponding to a local switching half-width of approximately 1.7 V and a pronounced positive imprint of approximately +1.8 V. In addition, one of the two dominant phase-contrast states occupies approximately 92% of the analyzed area (phase-state population ≈ 92:8), compared with an almost balanced 48:52 population in the gas transport crystal. This strong bias in both the switching loop and the phase-state population provides quantitative evidence for a pinned internal-field state in the In-rich CIPS/IPS system.

A related strongly asymmetric domain population was reported for CIPS/In_4/3_P_2_S_6_ heterostructures with compositionally segregated chemical domains [13], although the detailed domain morphology is also sensitive to thermal history. The present response is consistent with In_4_/_3_P_2_S_6_-rich chemical regions, which are known to generate compressive strain in the CIPS component and therefore an effective positive chemical pressure [13,14,15]. The corresponding increase in TC and the stabilization of the polar free-energy minima favor a more rigid polarization configuration and stronger domain pinning. Thus, In enrichment stabilizes the ferrielectric state thermodynamically while simultaneously reducing the configurational freedom of the domain pattern.

### 5.3. Unified Picture of Composition, Growth Defects, and Domain Organization

The PFM results reveal two complementary and partially coupled mechanisms controlling the domain structure of CIPS. Stoichiometric deviation modifies the thermodynamic landscape through composition-dependent strain, Cu-site occupancy, and interlayer coupling. Cu enrichment moves the system toward a softer negative-pressure-like regime, whereas the In-enriched response is consistent with a positive chemical pressure contribution associated with CIPS/IPS phase segregation. The growth route, however, independently contributes through the density and spatial distribution of extended defects, internal fields, and domain-wall pinning centers. Because composition and synthesis conditions are not fully orthogonal variables in the present sample set, these contributions cannot be quantitatively separated in all cases, particularly for the Bridgman–Stockbarger crystal.

These mechanisms are not independent. A local change in the Cu-ion potential landscape determines how readily a polar region can reconfigure, whereas growth defects and chemical interfaces determine where such reconfiguration is spatially favored or inhibited. The resulting domain morphology is therefore a mesoscale manifestation of the same microscopic variables that control the dielectric anomaly and Raman response: Cu-ion configurational freedom, interlayer coupling, strain, and defect-mediated pinning. This correspondence links the structural, dielectric, vibrational, and PFM observations within a single physical picture and provides the basis for the conclusions drawn below.

The observed differences in domain morphology are consistent with the unconventional switching dynamics of CIPS, in which polarization reversal is mediated primarily by single Cu-dipole flips rather than by classical domain wall motion. Consequently, local changes in the Cu-ion energy landscape (induced by stoichiometry or growth defects) strongly influence both the mobility of polar regions and the effectiveness of pinning centers. The quantitative switching and phase-state parameters are summarized in Table 3.

The observed differences in domain morphology are also consistent with the multi-pathway nature of Cu-ion dynamics in CIPS. Cu-ion migration proceeds via two distinct channels: low-barrier intralayer hopping (primarily responsible for polarization switching) and higher-barrier interlayer hopping across the van der Waals gap (responsible for long-range ionic conductivity). Density functional calculations yield characteristic migration barriers of approximately 0.05 eV for intralayer Cu1–Cu2 hopping, 0.7 eV for the polar Cu1u–Cu1d flip, and 1.1 eV for interlayer migration [49]. Experimental activation energies extracted from ionic conductivity (0.6–0.7 eV) are consistent with the higher of the intralayer barriers and a partial contribution of interlayer processes [4,32]. Stoichiometric deviations and growth-induced defects modify the relative accessibility of these pathways, thereby coupling the Cu-ion configurational landscape to the observed shifts in TC, dielectric response and domain morphology.

From an application perspective, the ability to tune local switching thresholds, imprint, and domain state stability through stoichiometry and growth conditions is directly relevant to non-volatile ferroelectric memory. Recent work has demonstrated non-volatile polarization switching in CIPS enabled by imprint field engineering [50], while CIPS-containing hybrid ferroelectrics have been used to realize ambipolar non-volatile MoS_2_ memory transistors [51]. Although retention and endurance are not measured in the present study, our results identify stoichiometry- and growth-controlled internal bias, switching energetics, and domain pinning as additional materials-level parameters for tailoring CIPS-based memory functionality.

## 6. Conclusions

The present study demonstrates that the ferrielectric properties of CuInP_2_S_6_ are highly sensitive to both deviations from stoichiometry and the defect landscape introduced by crystal growth conditions. Thus, the composition and synthesis route should be regarded as coupled but distinct control parameters rather than as interchangeable origins of the observed behavior. Their combined control of transition temperature, local switching bias, and domain pinning is particularly relevant for the materials-level optimization of CIPS-based non-volatile devices. The systematic comparison of Bridgman–Stockbarger, gas transport, Cu-enriched and In-enriched crystals reveals consistent shifts in transition temperature, dielectric response and mesoscale domain morphology. These observations are unified by a common microscopic picture based on composition- and growth-dependent changes in Cu-ion configurational freedom, interlayer coupling, strain and defect-mediated pinning. In enrichment shifts the ferrielectric transition to higher temperature and is consistent with the formation of CIPS/In_4_/_3_P_2_S_6_ chemical heterostructures. The associated lattice mismatch produces an effective positive chemical pressure on the CIPS regions, stabilizing the polar state and favoring a rigid, strongly biased domain configuration. Cu enrichment produces the opposite trend: lower *T_C_*, enhanced dielectric loss and a more fragmented domain state, all of which are characteristic of a negative-pressure-like softening of the Cu-ion energy landscape. This stoichiometry–pressure correspondence is supported by uniaxial pressure measurements, the multiwell Landau–Ginzburg–Devonshire description and first-principles comparisons of bulk and weakly coupled single-layer CIPS. The analogy is phenomenological rather than strictly thermodynamic, yet both chemical and mechanical control parameters act on the same underlying degrees of freedom associated with Cu-ion configuration and interlayer interactions. Crystal growth conditions provide an independent control parameter. Gas transport crystals exhibit the most uniform domain organization and the smallest local imprint, whereas melt-grown crystals contain extended structural inhomogeneities that strongly influence domain wall pinning. Consequently, the observed PFM morphology is a mesoscale manifestation of the combined thermodynamic and defect landscapes. Overall, the results establish stoichiometry, internal chemical pressure and growth-induced defects as interconnected and experimentally accessible tools for tuning ferrielectric order in CuInP_2_S_6_. The demonstrated ability to systematically change the transition temperature, dielectric losses and domain morphology through stoichiometry and growth conditions offers a practical route toward optimizing CIPS for memristive, neuromorphic and non-volatile memory applications, where controlled Cu-ion dynamics and stable polar domain configurations are decisive for device performance and reliability.

## Figures and Tables

**Table 1:** EDS-derived averaged compositions and ferrielectric transition temperatures *T_C_* of the investigated CuInP_2_S_6_ crystals. The compositions represent averages of 5–7 measurements acquired at different sample locations.

Sample	EDS Composition	*T_C_* (K)
CuInP_2_S_6_ (gas transport)	Cu_1.02_In_1.04_P_2.03_S_6_	317
CuInP_2_S_6_ (Bridgman–Stockbarger)	Cu_1.10_In_0.93_P_2.04_S_6_	307
CuInP_2_S_6_ + Cu_2_S	Cu_1.05_In_0.97_P_2_S_6.01_	312
CuInP_2_S_6_ + In_2_S_3_	Cu_0.75_In_1.15_P_2_S_6.02_	322

**Table 2:** Average Mulliken charges calculated for bulk and single-layer (1L) CIPS.

Species	Bulk CIPS	1L CIPS
Cu	0.288	0.279
In	0.886	0.847
P	0.751	0.750
S	−0.446	−0.438

**Table 3:** Quantitative parameters extracted from local PFM switching loops and phase images of the investigated CuInP_2_S_6_ crystals. *V_sw_*^−^ and *V_sw_*^+^ denote the negative- and positive-branch local switching thresholds, respectively, determined from the minima of the PFM amplitude loops. The local switching half-width and imprint voltage were calculated as V_c_* = (*V_sw_*^+^ − *V_sw_*^−^)/2 and *V_imprint_* = (*V_sw_*^+^ + *V_sw_*^−^)/2, respectively. The phase-state population represents the relative area occupied by the two dominant PFM phase-contrast states; the absolute sign of the polarization is not assigned from the color contrast alone.

Sample	V_sw_^−^ (V)	V_sw_^+^ (V)	V_c_* (V)	V_imprint_ (V)	Phase-State Population A:B
Bridgman–Stockbarger	≈−4.7	≈+4.6	≈4.6	≈−0.1	≈56:44
Cu-enriched	≈−2.2	≈+1.7	≈2.0	≈−0.2	≈57:43
Gas transport	≈−4.7	≈+4.5	≈4.6	≈−0.1	≈48:52
In-enriched	≈+0.1	≈+3.5	≈1.7	≈+1.8	≈92:8

## Data Availability

The data presented in this study are available within the article and its Supplementary Materials. Additional datasets are available from the corresponding author upon reasonable request.

## Supplementary Materials

The following supporting information can be downloaded at: https://www.mdpi.com/article/10.3390/nano16211336/s1, Section S1: EDS Data; Figure S1: Representative SEM surface image and local EDS analysis (elemental quantification and spectrum) for gas-transport CIPS; Figure S2: Representative SEM surface image and local EDS analysis (elemental quantification and spectrum) for Bridgman–Stockbarger CIPS; Figure S3: Representative SEM surface image and local EDS analysis (elemental quantification and spectrum) for Cu-enriched CuInP_2_S_6_ + Cu_2_S; Figure S4: Representative SEM surface image and local EDS analysis (elemental quantification and spectrum) for In-enriched CuInP_2_S_6_ + In_2_S_3_. Pronounced domain-like contrast is visible in the SEM image, similar to the chemical-domain morphology reported in Ref. [13] of the main text. The quantitative EDS values correspond to a single representative analyzed area and should not be interpreted as the averaged composition of the sample; Section S2: Complete Dielectric Spectra; Figure S5: Temperature–frequency dependences of (a) the real (ε′) and (b) the imaginary (ε″) components of the complex dielectric permittivity (ε* =ε′ − iε″) for a Bridgman–Stockbarger-grown CuInP_2_S_6_ crystal; Figure S6: Temperature–frequency dependences of (a) the real (ε′) and (b) the imaginary (ε″) components of the complex dielectric permittivity (ε* = ε′ − iε″) for a Cu-enriched CuInP_2_S_6_ + Cu_2_S crystal grown by chemical vapor transport (CVT); Figure S7: Temperature–frequency dependences of (a) the real (ε′) and (b) the imaginary (ε″) components of the complex dielectric permittivity (ε* = ε′ − iε″) for a gas-transport-grown CuInP_2_S_6_ crystal; Figure S8: Temperature–frequency dependences of (a) the real (ε′) and (b) the imaginary (ε″) components of the complex dielectric permittivity (ε* = ε′ − iε″) for an In-enriched CuInP_2_S_6_ + In_2_S_3_ crystal grown by chemical vapor transport (CVT); Section S3: Landau–Ginzburg–Devonshire electrostriction coefficients of CuInP_2_S_6_. Temperature-dependent linear and nonlinear electrostriction coefficients used in the LGD analysis, together with a comparison to experimental data [52]; Section S4: Quantitative Raman parameters; Table S1: Quantitative Raman parameters. Each cell is reported as peak position ν (cm^−1^)/normalized intensity I/I_P–P_/FWHM (cm^−1^).
