# Supplementary material for: Linking Stoichiometry, Chemical Pressure, and Domain Structure in the van der Waals Ferrielectric CuInP2S6
Source: Nanomaterials (Basel). 2026 Oct 22;16(21):1336. doi: 10.3390/nano16211336 (PMC13764896; doi:10.3390/nano16211336)
Supplement: Supplementary file 1 [file nanomaterials-16-01336-s001.zip › nanomaterials-4625608-Supplementary.pdf]

Linking Stoichiometry, Chemical Pressure, and Domain Structure in the van der Waals Ferrielectric $CuInP_2S_6$

Dávid Gál [1,2], Sándor Lenk [3], István Rigó [1], Miklós Veres [1], László Péter [1], Anton Kohutych [4], Konstantin Glukhov [4], Nyomó Dominika [4] and Alexander Molnar [4,*]

[1] HUN-REN Wigner Research Centre for Physics, Konkoly-Thege Miklós út 29-33, 1121 Budapest, Hungary; gal.david@wigner.hun-ren.hu (D.G.); rigo.istvan@wigner.hu (I.R.); veres.miklos@wigner.hun-ren.hu (M.V.); peter.laszlo@wigner.hun-ren.hu (L.P.)
[2] Institute of Physics, University of Pécs, Ifjúság útja. 6, 7624 Pécs, Hungary
[3] Department of Atomic Physics, Institute of Physics, Budapest University of Technology and Economics, Műegyetem rkp. 3, 1111 Budapest, Hungary; lenk.sandor@ttk.bme.hu
[4] Department of the Physics of Semiconductors, Uzhhorod National University, 54 Voloshina Str., 88000 Uzhhorod, Ukraine; anton.kohutych@uzhnu.edu.ua (A.K.); konstantin.glukhov@uzhnu.edu.ua (K.G.)
* Correspondence: alex.molnar@uzhnu.edu.ua

S1. EDS Data

The EDS spectra presented in Figures S1–S4 correspond to representative local measurements. The compositions reported in Table 1 of the main text were obtained by averaging 5–7 measurements acquired at different locations of each sample. For the representative spectra shown here, the EDAX APEX eZAF quantification reports overall analysis uncertainties of 8.40–9.28%, with element-specific estimated errors of approximately 3.4% for Cu and 4.7–4.8% for In. These quantities characterize the analytical uncertainty of the EDS quantification and should not be interpreted as point-to-point standard deviations. The local spectra are shown as representative measurements, whereas Table 1 reports the sample-averaged EDS compositions used in the composition-dependent analysis.

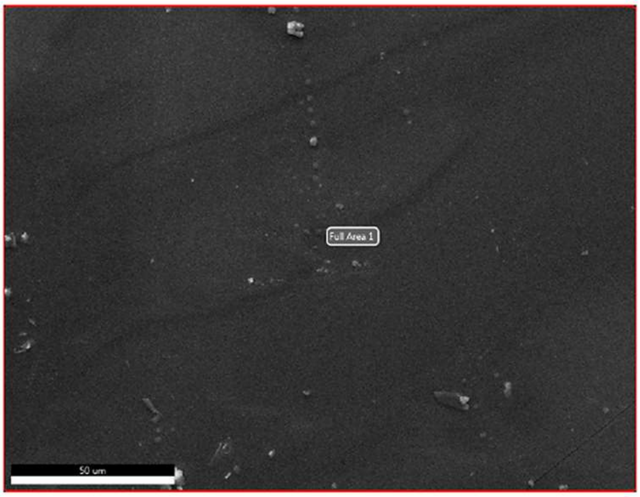

eZAF Quant Result - Analysis Uncertainty: 8.84 %

Element	Weight %	MDL	Atomic %	Error %
P K	14.76	0.19	20.68	5.04
S K	43.70	0.22	59.16	4.81
Cu K	14.61	1.78	9.98	3.46
In L	26.93	0.90	10.18	4.81

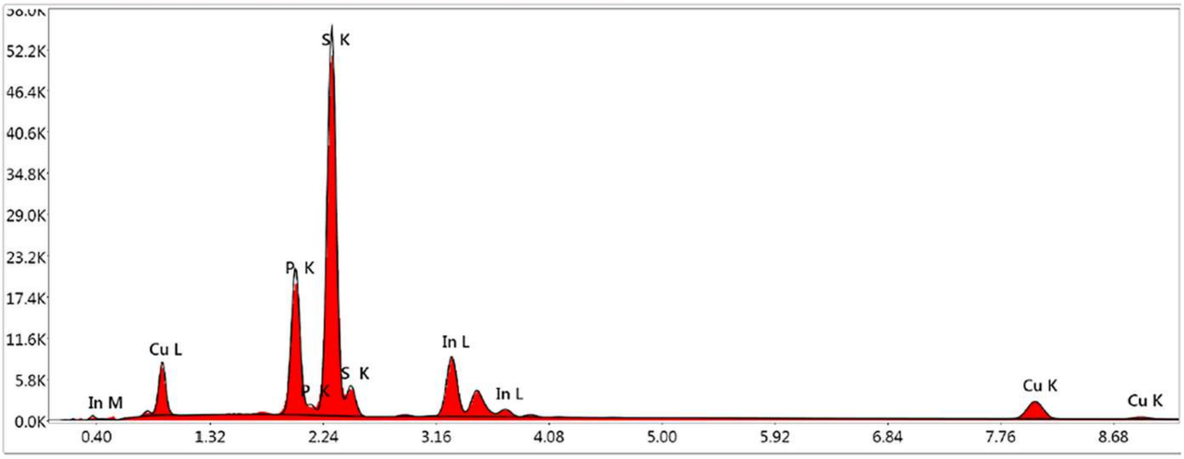

Figure S1. Representative SEM surface image and local EDS analysis (elemental quantification and spectrum) for gas-transport CIPS.

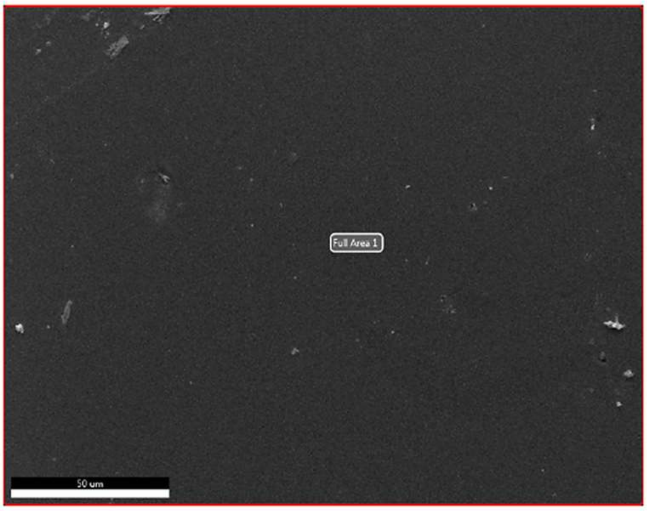

eZAF Quant Result - Analysis Uncertainty: 9.28 %

Element	Weight %	MDL	Atomic %	Error %
P K	14.67	0.20	20.59	5.07
S K	43.40	0.23	58.85	4.82
Cu K	15.31	1.84	10.47	3.40
In L	26.62	0.92	10.08	4.82

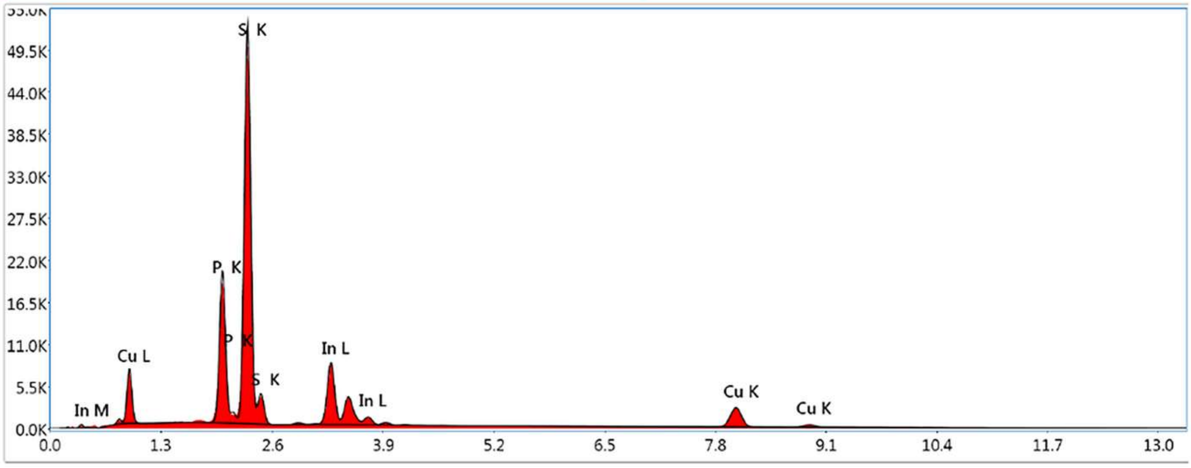

Figure S2. Representative SEM surface image and local EDS analysis (elemental quantification and spectrum) for Bridgman–Stockbarger CIPS.

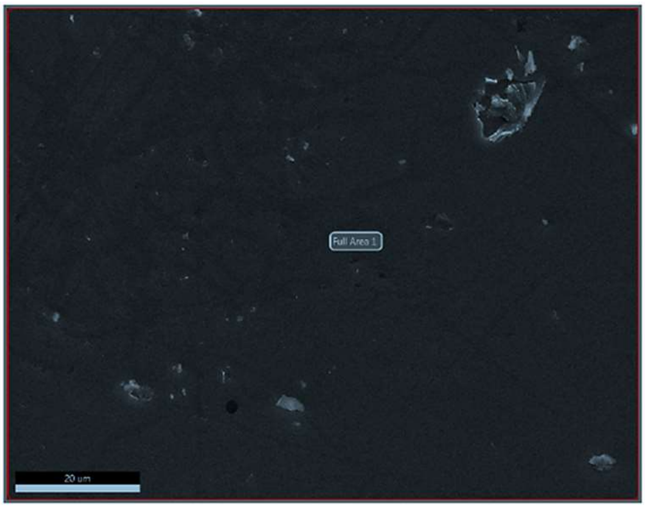

eZAF Quant Result - Analysis Uncertainty: 8.79 %

Element	Weight %	MDL	Atomic %	Error %
P K	14.57	0.20	20.49	5.08
S K	43.25	0.23	58.77	4.82
Cu K	15.42	1.83	10.57	3.40
In L	26.77	0.92	10.16	4.78

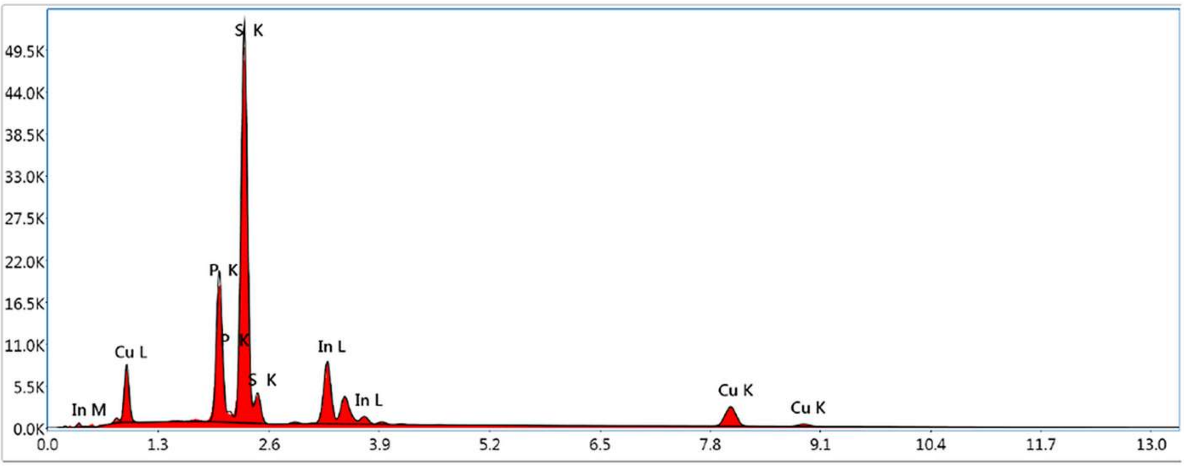

Figure S3. Representative SEM surface image and local EDS analysis (elemental quantification and spectrum) for Cu-enriched $CuInP_2S_6$ + Cu_2S.

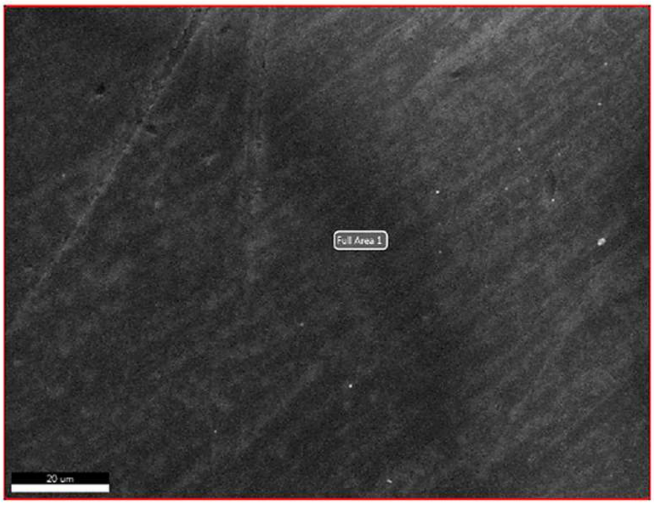

eZAF Quant Result - Analysis Uncertainty: 8.40 %

Element	Weight %	MDL	Atomic %	Error %
P K	14.79	0.20	20.77	5.04
S K	43.62	0.22	59.16	4.81
Cu K	14.15	1.82	9.68	3.49
In L	27.44	0.91	10.39	4.72

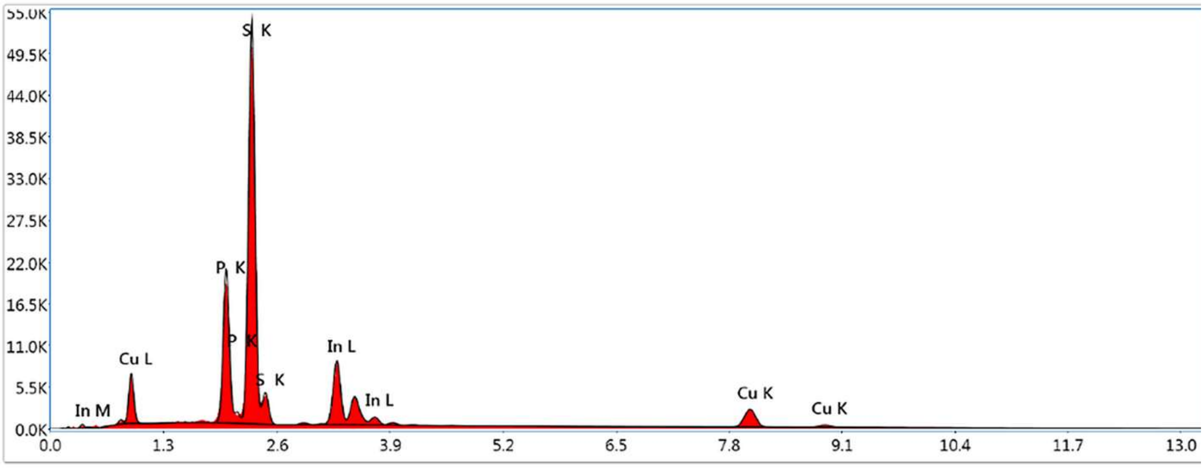

Figure S4. Representative SEM surface image and local EDS analysis (elemental quantification and spectrum) for In-enriched $CuInP_2S_6$ + In_2S_3. Pronounced domain-like contrast is visible in the SEM image, similar to the chemical-domain morphology reported in Ref. [13] of the main text. The quantitative EDS values correspond to a single representative analyzed area and should not be interpreted as the averaged composition of the sample.

S2. Complete Dielectric Spectra

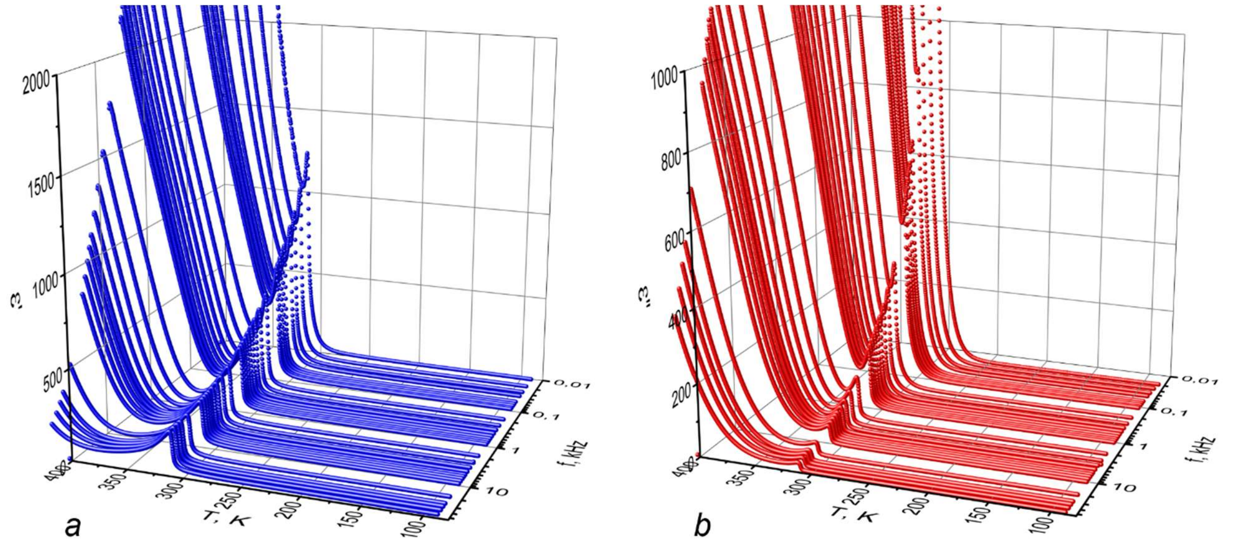

Figure S5. Temperature–frequency dependences of (*a*) the real, ε′, and (*b*) imaginary, ε″, components of the complex dielectric permittivity, ε* = ε′ − iε″, for a Bridgman–Stockbarger-grown $CuInP_2S_6$ crystal.

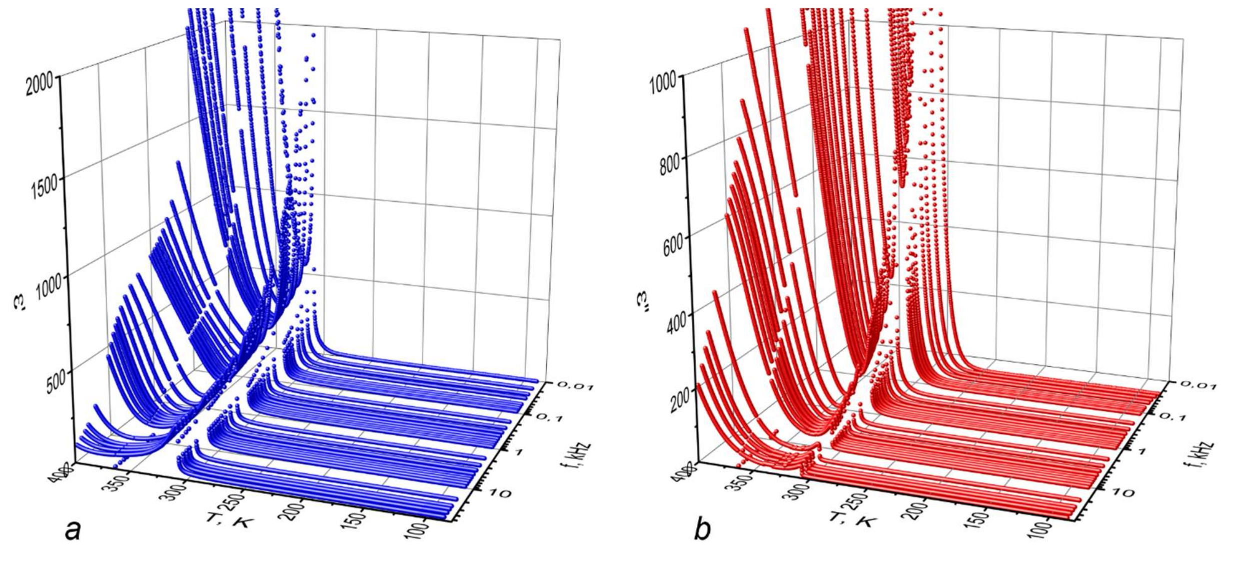

Figure S6. Temperature–frequency dependences of (a) the real, ε′, and (b) imaginary, ε″, components of the complex dielectric permittivity, ε* = ε′ − iε″, for a Cu-enriched $CuInP_2S_6$ + Cu_2S crystal grown by chemical vapor transport (CVT).

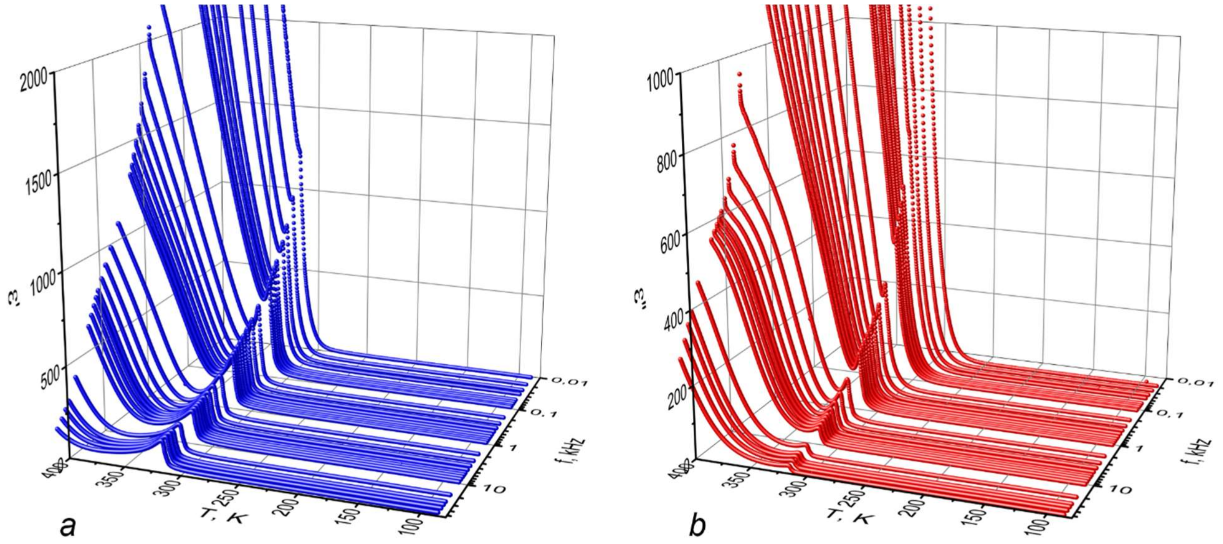

Figure S7. Temperature–frequency dependences of (*a*) the real, ε′, and (*b*) imaginary, ε″, components of the complex dielectric permittivity, ε* = ε′ − iε″, for a gas-transport-grown $CuInP_2S_6$ crystal.

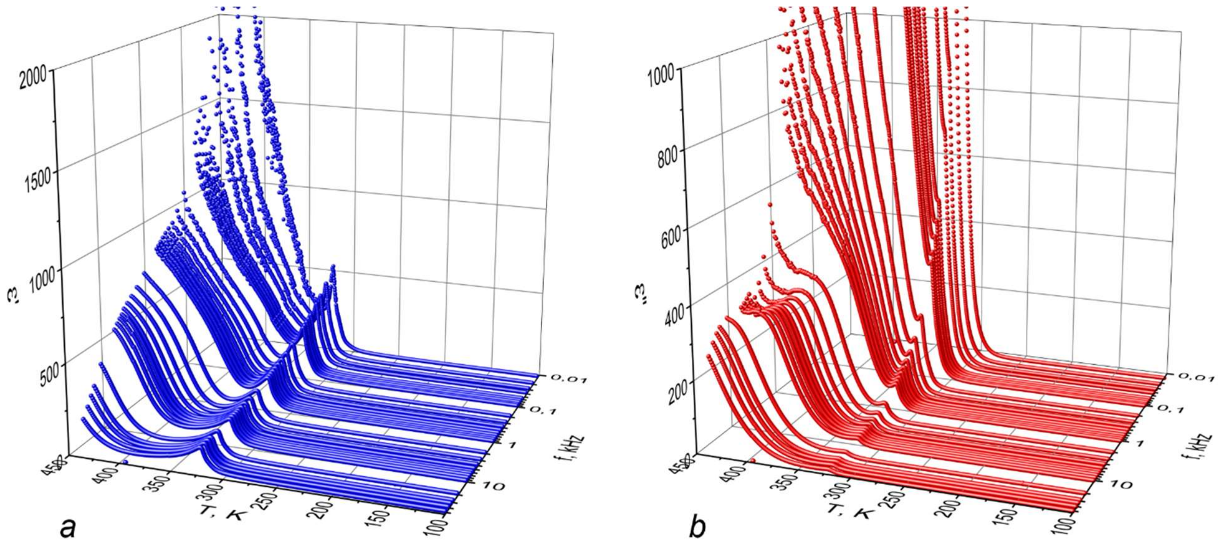

Figure S8. Temperature–frequency dependences of (a) the real, ε′, and (b) imaginary, ε″, components of the complex dielectric permittivity, ε* = ε′ − iε″, for an In-enriched $CuInP_2S_6 + In_2S_3$ crystal grown by chemical vapor transport (CVT).

S3. Landau–Ginzburg–Devonshire electrostriction coefficients of $CuInP_2S_6$

The linear (Q_{i3}) and nonlinear (Z_{i33}) electrostriction coefficients used in the LGD analysis of the main text were taken from the thermodynamic potential reconstructed by Morozovska et al. [38]. They exhibit a pronounced linear temperature dependence (T in kelvin):

Linear electrostriction coefficients ($m^4\ C^{-2}$):

$$Q_{13} = 1.70136 - 0.00363T,$$

$$Q_{23} = 1.13424 - 0.00242T,$$

$$Q_{33} = -5.622 + 0.0105T.$$

Nonlinear electrostriction coefficients ($m^4\ C^{-2}$):

$$Z_{133} = -2059.65 + 0.8T,$$

$$Z_{233} = -1211.26 + 0.45T,$$

$$Z_{333} = 1381.37 - 12T.$$

At room temperature (T=295 K) these expressions yield approximately $Q_{33} \approx -2.5$ $m^4\ C^{-2}$ and $Z_{333} \approx -2160$ $m^4\ C^{-2}$. The negative sign of Q_{33} is consistent with independent experimental determinations of the longitudinal electrostriction coefficient of bulk CIPS ($Q_{33} \approx -3.2$ to -3.4 $m^4\ C^{-2}$) [52]. The strong temperature dependence and the unconventional signs of both Q_{i3} and Z_{i33} are essential for the appearance of a flattened multiwell free-energy landscape under tensile (negative-pressure-like) conditions, which underpins the stoichiometry–pressure correspondence discussed in the main text.

S4. Quantitative Raman parameters.

Selected Raman bands were fitted using identical pseudo-Voigt profiles with a local linear background for all four samples. The table below summarizes the fitted peak positions, intensities normalized to the fitted P–P-related band, and FWHM values. The same spectral windows and fitting procedure were used for all samples to enable direct comparison

Table S1. Quantitative Raman parameters. Each cell is reported as peak position ν (cm^{-1}) / normalized intensity $I/I_{P\text{–}P}$ / FWHM (cm^{-1}).

Approx. mode	Bridgman–Stockbarger	Gas-transport (CTR)	Cu-enriched	In-enriched
~70 cm^{-1}	71.15 / 0.132 / 6.26	70.78 / 0.165 / 7.42	70.11 / 0.203 / 6.43	69.50 / 0.170 / 7.71
~104 cm^{-1}	104.96 / 0.521 / 6.73	103.90 / 0.480 / 7.39	103.68 / 0.503 / 6.43	103.22 / 0.511 / 7.68
~161 cm^{-1}	162.08 / 0.127 / 11.31	161.18 / 0.125 / 12.85	160.84 / 0.133 / 11.09	160.41 / 0.148 / 11.65
~264 cm^{-1}	264.45 / 0.664 / 13.02	264.40 / 0.729 / 14.57	263.48 / 0.682 / 13.15	263.58 / 0.789 / 14.85
~317 cm^{-1}	317.78 / 0.283 / 13.81	316.78 / 0.364 / 14.00	316.61 / 0.439 / 13.93	316.07 / 0.394 / 13.36
P–P ~374 cm^{-1}	375.12 / 1.000 / 6.65	374.69 / 1.000 / 7.17	374.01 / 1.000 / 6.58	373.67 / 1.000 / 7.43
~558 cm^{-1}	558.54 / 0.230 / 6.84	557.68 / 0.236 / 8.09	557.09 / 0.219 / 7.47	556.30 / 0.230 / 9.43
